# Anionic Effect on Electrical Transport Properties of Solid Co^2+/3+^ Redox Mediators

**DOI:** 10.3390/polym16101436

**Published:** 2024-05-19

**Authors:** Ravindra Kumar Gupta, Ahamad Imran, Aslam Khan

**Affiliations:** King Abdullah Institute for Nanotechnology, King Saud University, Riyadh 11451, Saudi Arabia; aimran@ksu.edu.sa (A.I.); aslamkhan@ksu.edu.sa (A.K.)

**Keywords:** dye-sensitized solar cells, solid electrolytes, PEO, electrical conductivity, anionic effect

## Abstract

In a solid-state dye-sensitized solar cell, a fast-ion conducting (σ_25°C_ > 10^−4^ S cm^−1^) solid redox mediator (SRM; electrolyte) helps in fast dye regeneration and back-electron transfer inhibition. In this work, we synthesized solid Co^2+/3+^ redox mediators using a [(1 − *x*)succinonitrile: *x* poly(ethylene oxide)] matrix, LiX, Co(tris-2,2′-bipyridine)_3_(bis(trifluoromethyl) sulfonylimide)_2_, and Co(tris-2,2′-bipyridine)_3_(bis(trifluoromethyl) sulfonylimide)_3_ via the solution-cast method, and the results were compared with those of their acetonitrile-based liquid counterparts. The notation *x* is a weight fraction (=0, 0.5, and 1), and X represents an anion. The anion was either bis(trifluoromethyl) sulfonylimide [TFSI^−^; ionic size, 0.79 nm] or trifluoromethanesulfonate [Triflate^−^; ionic size, 0.44 nm]. The delocalized electrons and a low value of lattice energy for the anions made the lithium salts highly dissociable in the matrix. The electrolytes exhibited σ_25°C_ ≈ 2.1 × 10^−3^ (1.5 × 10^−3^), 7.2 × 10^−4^ (3.1 × 10^−4^), and 9.7 × 10^−7^ (6.3 × 10^−7^) S cm^−1^ for *x* = 0, 0.5, and 1, respectively, with X = TFSI^−^ (Triflate^−^) ions. The log σ–*T*^−1^ plot portrayed a linear curve for *x* = 0 and 1, and a downward curve for *x* = 0.5. The electrical transport study showed σ(TFSI^−^) > σ(Triflate^−^), with lower activation energy for TFSI^−^ ions. The anionic effect increased from *x* = 0 to 1. This effect was explained using conventional techniques, such as Fourier transform infrared spectroscopy (FT-IR), X-ray diffractometry (XRD), X-ray photoelectron spectroscopy (XPS), scanning electron microscopy (SEM), UV–visible spectroscopy (UV-vis), differential scanning calorimetry (DSC), and thermogravimetric analysis (TGA).

## 1. Introduction

Solar cells convert sunlight energy into electrical energy without producing harmful greenhouse gases, which is one of the crucial factors in climate change [1,2]. Amongst various types of solar cells, only solid-state dye-sensitized solar cells (DSSCs) are beneficial in high-temperature regions such as Gulf countries [3]. The conversion efficiency decreases in the high-temperature region and when there is a low angle of sunlight incidence on the solar cells, except for DSSCs [1,2]. The exception occurs due to redox mediators (electrolytes). The solid nature of a redox mediator makes DSSCs scalable in manufacturing and eliminates the odds of a liquid or gel counterpart. The electrolyte participates in the following redox reactions: oxidation at the working electrode for dye regeneration, and reduction of the oxidized ions at the counter electrode. The reaction speed at the electrolyte/electrode interface largely controls DSSC efficiency. The speed depends on the electrical conductivity (σ) of the redox mediator. Therefore, a solid redox mediator (SRM) with σ_25°C_ greater than 10^−4^ S cm^−1^ is highly desirable for the fast movement of the redox couple. For a review, see references [4,5].

Solvent-free solid electrolytes with a redox couple of $I^{-}/I_{3}^{-}$ have been known about since 2002 [6,7]. These electrolytes generally have the form of a matrix (-plasticizer)-MI-I_2_, where M represents a cation [6,7,8,9,10,11,12,13,14,15,16,17,18,19]. The matrix is generally a solid polymer, e.g., poly(ethylene oxide), abbreviated as PEO [6,7,10], or a plastic crystal, e.g., succinonitrile (SN) [20,21]. Table S1 in the Supplementary Information lists all used abbreviations. Plasticizers, such as low-molecular-weight polymers [22,23,24,25], synthetic resins [26,27], ionic liquids [28,29,30], quantum dots [31], inorganic insulators [6,7], biopolymers [13], plastic crystals [32,33,34,35,36,37], and nanoclays [38], have been applied with PEO to decrease its crystallinity. Cations are either inorganic [28,29,30] or organic [20,39]. It was also observed that the σ-value increases with increasing size of the cation, revealing its plasticizing property [39,40]. 

Recently, Gupta et al. [3] synthesized the first solid Co^2+/3+^ redox mediator. They utilized [(1 − *x*)SN: *x*PEO] as a matrix with *x* = 0, 0.5, and 1 in a weight fraction. Li-bis(trifluoromethyl) sulfonylimide was used as a source of Li^+^ ions. As a Co^2+^/Co^3+^ source, a mix of Co(tris-2,2′-bipyridine)_3_(bis(trifluoromethyl) sulfonylimide)_2_ and Co(tris-2,2′-bipyridine)_3_ (bis(trifluoromethyl) sulfonylimide)_3_ was employed. The bis(trifluoromethyl) sulfonylimide ion or (CF_3_SO_2_)_2_N^−^ is commonly abbreviated to TFSI^−^. The ligand, tris-(2,2′-bipyridine), shortened generally to bpy, binds the central cobalt ion. Figure S1 shows the chemical structure of ionic salts and matrices for comparison. As mentioned earlier, both SN and PEO are very good solid matrices, offering nitrile and ether, respectively, for ion transport in addition to the dissolution and complexation of ionic salt [32,33,34,35]. Specifically, SN is highly attractive because it acts as a solid solvent or plasticizer due to its low melting temperature (*T*_m_~58 °C), high dielectric constant (55 at 25 °C and 62.6 at 58 °C), high molar enthalpy (139.7 kJ mol^−1^), and high donor number (14 kcal mol^−1^ at 25 °C) [41]. LiTFSI provides Li^+^ ions, boosting dye regeneration and, thereby, the photocurrent. The TFSI^−^ ion has delocalized electrons with a low value of lattice energy, which increases the dissociability of ionic salt in a solvent or polymer [42]. This anion also has a large ionic size of 0.79 nm [43] and low ionic mobility [42], making it less contributory to the total electrical conductivity. The cobalt salts Co(bpy)_3_(TFSI)_2_ and Co(bpy)_3_(TFSI)_3_ provide Co^2+^ and Co^3+^ redox species, respectively [44]. Owing to the large size of Co^2+^ (0.13 nm) and Co^3+^ (0.11 nm) ions [41], accompanied by the excellent plasticizing properties of TFSI^−^ ions and succinonitrile, SRMs achieved σ_25°C_ of 2.1 × 10^−3^ S cm^−1^ for *x* = 0, 7.2 × 10^−4^ S cm^−1^ for *x* = 0.5, and 9.7 × 10^−7^ S cm^−1^ for *x* = 1, compared with σ_25°C_ of 1.7 × 10^−2^ S cm^−1^ for acetonitrile (ACN)-based liquid redox mediators (LRMs). 

LiCF_3_SO_3_ is one of the lithium salts that is highly used for preparing solid polymer electrolytes for lithium-ion batteries [45]. The anion CF_3_SO_3_^−^ is generally written as Triflate^−^ for trifluoromethanesulfonic acid. Similar to the TFSI^−^ ion, this anion coordinates weakly with a cation and therefore does not allow ion pairing [46]. This anion has an ionic size of 0.44 nm, which is smaller to that of the TFSI^−^ ion. LiTriflate possesses higher values of donor number and ionic mobility, and lower values of molecular weight and dissociation constant, than LiTFSI. LiTriflate, therefore, possesses a lower value of σ_25°C_ in a mixture of solvents [42]. In this study, we replaced the LiTFSI of [(1 − *x*)SN: *x*PEO]-LiTFSI-Co salts with LiTriflate to show the anionic effect on the electrical transport properties of SRMs. As previously mentioned, *x* = 0, 0.5, and 1 in the weight fraction. Hereafter, lithium salt is represented by LiX, where X = TFSI^−^ or Triflate^−^. The molar composition and preparatory methods for the SRM [(1 − *x*)SN: *x*PEO]-LiX-Co salts are identical. We also prepared ACN-based liquid counterparts (LRMs) identically, as reported by Mathew et al. [44], for comparison. The preparation of the SRMs with *x* = 0 was identical to that of the LRMs. We used the conventional method (solution-cast) of preparation for the PEO-based SRMs (*x* = 0.5 and 1). The anionic effect on the electrical transport properties was explained using Fourier transform infrared spectroscopy (FT-IR), X-ray diffractometry (XRD), X-ray photoelectron spectroscopy (XPS), scanning electron microscopy (SEM), UV–visible spectroscopy (UV-vis), differential scanning calorimetry (DSC), and thermogravimetric analysis (TGA). 

## 2. Materials and Methods

The highly pure chemicals (*cf*. Table S2) were purchased and used without purification. The ACN-based LRMs and SN-based SRMs were synthesized using 0.1 M LiX, 0.25 M Co(bpy)_3_(TFSI)_2_, and 0.06 M Co(bpy)_3_(TFSI)_3_ in ACN and SN, respectively, under stirring at 65 °C for 24 h [3,44]. The PEO-based SRMs with *x* = 0.5 and 1 were synthesized using the solution-cast method [32,33,34,35]. The SRMs with *x* = 1 underwent a complete replacement of SN with PEO, followed by rigorous stirring in acetonitrile at 65 °C for 48 h, casting on a Teflon Petri dish, and drying under a nitrogen gas atmosphere at room temperature. The SRMs with *x* = 0.5 were synthesized similarly. The redox mediators were characterized using conventional techniques, such as impedance spectroscopy (IS), FT-IR, XRD, XPS, SEM, UV-vis, DSC, and TGA, which are described in the Supplementary Information (*cf.* Table S3) [3,47]. Table S4 lists the equipment used for the measurements. 

## 3. Results and Discussion

**Figure 1:**
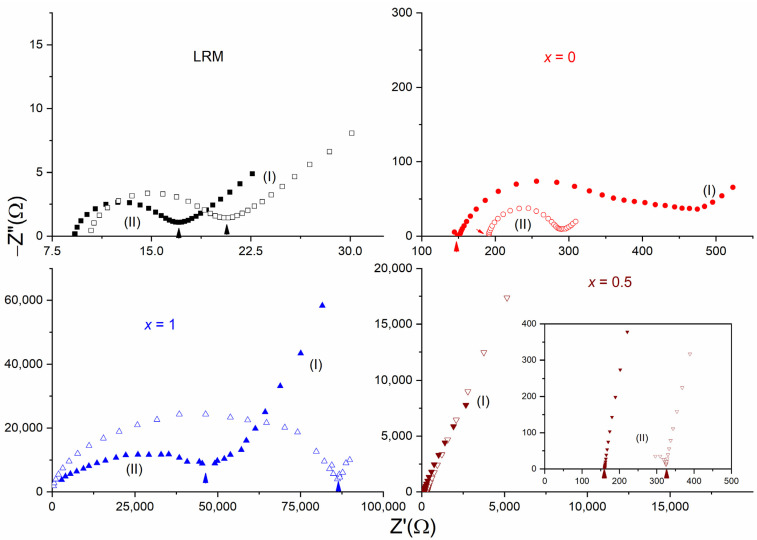
Nyquist curves of SRM [(1 − *x*)SN: *x*PEO]-LiX-Co salts (*x* = 0, 0.5, and 1) along with their liquid counterparts (LRMs) at 25 °C for anions, X = TFSI^−^ (solid symbols), and Triflate^−^ (open symbols). (I) and (II) correspond to low- and high-frequency regions, respectively.

Impedance spectroscopy is a tool used to study the electrical transport properties of an electrolyte [48,49]. A complex impedance plot, widely known as the Nyquist plot, helps to deduce bulk resistance (*R*_b_) and, thereby, the electrical conductivity of the electrolyte. Figure 1 shows Nyquist curves of the SRM [(1 − *x*)SN: *x*PEO]-LiX-Co salts along with their liquid counterparts (LRMs) at 25 °C. Here, *x* = 0, 0.5, and 1, and X = TFSI^−^ and Triflate^−^. In this figure, region (I) indicates a linear trend in the low-frequency domain because of the blocking-electrode effect, and region (II) corresponds to a semi-circle in the high-frequency domain because of the ionic diffusion effect [48,49]. The Nyquist curve can be fitted using an equivalent circuit [49], *R*_s,I_ + (*R*_b_‖*C*_1_)_I_ + *C*_2,II_, where the notations have their usual meaning. For example, *R*_s_ stands for series resistance due to leads, *R*_b_ for bulk resistance, *C*_1_ for chemical capacitance, and *C*_2_ for double-layer capacitance. The LRMs exhibited nearly identical and perfect trends: a linear curve in region (I) and a semi-circle in region (II). Having a plastic crystal phase, the SRMs with *x* = 0 had a semi-circle similar to those of SN-LiI-I_2_ [21], but with a less prominent blocking-electrode effect. In addition, the semi-circle was slightly suppressed relative to those of the LRM-based redox mediators. The SRMs with *x* = 1 showed largely suppressed semi-circles, most probably because of the semi-crystalline nature of the PEO [50]. The SRMs with *x* = 0.5 portrayed region (I) only. We found a similar pattern for the (SN-PEO)-MI-I_2_ (M = Li^+^ or K^+^) SRMs [34,35]. This indicated the formation of amorphous domains by the short and entangled polymer chains induced by the plasticizing properties of succinonitrile [51,52]. Figure 1 also portrays the anionic effect. Relative to Triflate^−^, TFSI^−^ resulted in a low value of bulk resistance, as marked by an arrow. This was associated with a smaller semi-circle and a prominent linear trend, except for the redox mediator with *x* = 0.

We evaluated the σ_25°C_ values of liquid and solid redox mediators using the values of the *R_b_*, thickness, and area of the electrolyte. Table 1 lists the average values of σ_25°C_. The LRMs achieved σ_25°C_ ≈ 1.7 × 10^−2^ S cm^−1^ for X = TFSI^−^ and ≈ 1.6 × 10^−2^ S cm^−1^ for X = Triflate^−^, as reported earlier for liquid electrolytes [42]. We recently attained σ_25°C_ ≈10^−3^ S cm^−1^ for SN-LiI-I_2_, because of the solid solvent nature of SN [21]. We achieved similar σ_25°C_ values, ≈2.1 × 10^−3^ S cm^−1^ for X = TFSI^−^ and ≈1.5 × 10^−3^ S cm^−1^ for X = Triflate^−^ for the SRMs with *x* = 0. Compared with the LRMs, these values are less than an order of magnitude. The complete replacement of SN by PEO decreased the σ_25°C_ value to ≈9.7 × 10^−7^ S cm^−1^ for X = TFSI^−^ and ≈6.3 × 10^−7^ S cm^−1^ for X = Triflate^−^, which are considerably less than three orders of magnitude. A poor σ_25°C_ value was observed previously for several PEO-based solid $I^{-}/I_{3}^{-}$ redox mediators and occurred due to high PEO crystallinity, hindering ion transport [34,35]. The redox mediators with *x* = 0.5 achieved a σ_25°C_ value of ≈7.2 × 10^−4^ S cm^−1^ for X = TFSI^−^ and ≈3.1 × 10^−4^ S cm^−1^ for X = Triflate^−^. These values are more than two orders of magnitude higher than those of the redox mediators with *x* = 1 and nearly an order of magnitude lower than those of the redox mediators with *x* = 0. We found similar values (3–7 × 10^−4^ S cm^−1^) for the (PEO-SN) Blend-MI-I_2_ (M = Li^+^ and K^+^) SRMs, too [34,35]. This is due to the plasticizing nature of SN, which offers more amorphous regions for ion transport. One can note that TFSI^−^ resulted in a better σ_25°C_ value than Triflate^−^, specifically for the PEO-based redox mediators. This is due to several factors, such as the large ionic size; lower values of lattice energy, donor numbers, and ionic mobility; and higher values of the molecular weight and dissociation constant of TFSI^−^ [42]. 

**Figure 2:**
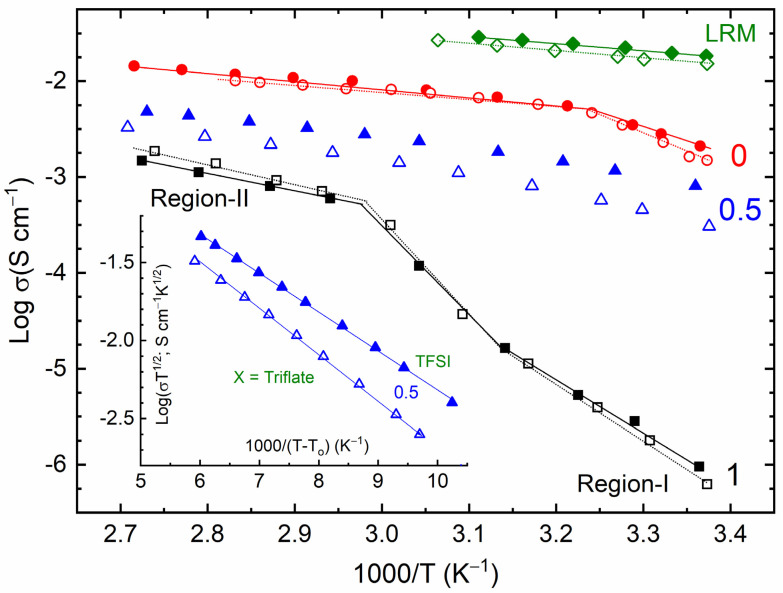
Log σ vs. *T*^−1^ curves of SRM [(1 − *x*)SN: *x*PEO]-LiX-Co salts with *x* = 0, 0.5, and 1. Inset: Vogel–Tamman–Fulcher (VTF) plots for SRMs with *x* = 0.5. X = TFSI^−^ ions (filled symbols) and Triflate^−^ ions (open symbols). LRMs, ACN-based liquid counterparts. For regions I and II, please see the text.

Figure 2 shows the log σ − *T*^−1^ curves of the SRM [(1 − x)SN: *x*PEO]-LiX-Co salts (*x* = 0, 0.5, and 1; X = TFSI^−^ or Triflate^−^) and their liquid counterparts (LRMs). The SRMs with *x* = 0 and 1 showed a linear trend similar to the LRMs, SN-LiI-I_2_, and PEO-KI-I_2_, revealing Arrhenius-type behavior of ion transport in the form of σ = σ_o_ exp[–*E*_a_/k_B_*T*] with the help of molecules or polymeric chains, where σ_o_, *E*_a_, and k_B_ are the pre-exponential factor, activation energy, and Boltzmann constant, respectively [21,32,35]. The SRMs with *x* = 0.5, on the other hand, had downward curves, just like the Blend-MI-I_2_. This showed Vogel–Tamman–Fulcher (VTF)-type behavior because of the formation of the amorphous phase. The VTF-type trend is expressed as σ = σ_o_*T*^−½^ exp[–*B*/k_B_(*T* − T_o_)], where *B* and T_o_ are the pseudo-activation energy and free-volume temperature, respectively. The inset of Figure 2 exhibits a linear log(σ*T*^½^) – (*T* − T_o_)^−1^ curve for SRMs with *x* = 0.5. We summarized the nature of redox mediators in Table 1. We also evaluated the values of *E*_a_ and *B* from the slopes of the linear curves of redox mediators and listed them in Table 1 for comparison. In this table, regions I and II correspond to the temperatures before and after the melting temperature of SRMs with *x* = 0 and 1. Both the SRMs, *x* = 0 and 1, possessed *E*_a_-values of more than 0.3 eV in the solid-state region (region-I), making them useless for device applications [53]. In contrast, the SRMs with *x* = 0.5 possessed quite a low *B*-value ≈ 0.06 eV, which was similar to those of Blend-MI-I_2_ [34,35]. Table 1 also shows lower activation energy values for the TFSI^−^-based redox mediators. As mentioned earlier, this is due to the several beneficial properties of TFSI^−^. 

**Figure 3:**
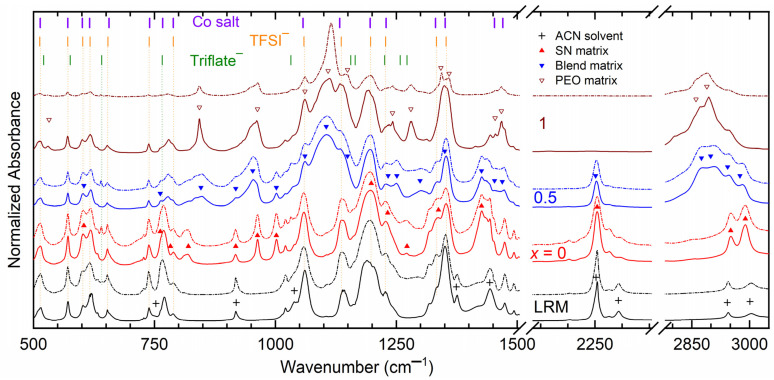
FT-IR spectra of SRM [(1 − *x*)SN: *x*PEO]-LiX-Co salts (*x* = 0, 0.5, and 1). LRM, ACN-based liquid counterpart. X = TFSI^−^ (solid lines) or Triflate^−^ (dotted lines). This also includes vibrational peaks of matrices, solvent, and ionic salts (vertical lines).

FT-IR spectroscopy is a tool for understanding matrix–salt interactions [3,34,35]. Interestingly, all the ingredients of the solid and liquid redox mediators are IR-active. Figure 3 shows the FT-IR spectra of SRM [(1 − *x*)SN: *x*PEO]-LiX-Co salts with *x* = 0, 0.5, and 1 and their liquid counterparts (LRMs) for X = TFSI^−^ and Triflate^−^. This also shows vibrational peaks of the matrices, solvent, and ionic salts [3,54]. The vibrational peaks of TFSI^−^ with their assignments are as follows: 513 and 571 cm^−1^ (δ_a,SO2_), 602 cm^−1^ (δ_a,SO2_), 654 cm^−1^ (δ_a,SO2_), 739 cm^−1^ (ν_s,SNS_), 789 cm^−1^ (ν_a,SNS_), 1059 cm^−1^ (ν_a,SNS_; free), 1136 cm^−1^ (ν_s,SO2_; free), 1197 cm^−1^ (ν_a,CF3_; free), 1228 cm^−1^ (ν_s,CF3_; paired), 1333 cm^−1^ (ν_a,SO2_; paired), and 1353 cm^−1^ (ν_a,SO2_; free) [55,56]. Triflate^−^ showed its presence through the peaks 521 cm^−1^ (δ_a,SO3_), 576 cm^−1^ (δ_a,SO3_), 641 cm^−1^ (δ_a,SO3_), 766 cm^−1^ (δ_s,CF3_), 1032 cm^−1^ (ν_s,SO3_; free), 1156 cm^−1^ (ν_a,CF3_; free), 1165 cm^−1^ (ν_a,CF3_; paired), 1226 cm^−1^ (ν_s,CF3_; free), 1258 cm^−1^ (ν_a,SO3_; paired), 1272 cm^−1^ (ν_a,SO3_; free), and 1300 cm^−1^ (paired) [57,58,59]. The cobalt salts have peaks of TFSI^−^ as well as a bpy ring structure (616, 767, 1229, 1453, and 1470 cm^−1^ [60]). Here, we used the notations ν for stretching, δ for bending, s for symmetric, and a for asymmetric mode. It is worth mentioning that the peak position of free ions remains unaltered with a change in the concentration of ionic salt [55,56]. We also noticed that several modes of TFSI^−^ merge with those of cobalt salts. Those of Triflate^−^ exhibited overlapping in several modes with those of TFSI^−^, too. 

FT-IR spectroscopy showed the least interaction between the solvent and salt for the LRMs. This was indicated by the least change in the positions of the modes for the solvent and salts, except at 739 cm^−1^ (753 cm^−1^ for ACN solvent) for both anions. Also, the ion pairing peak at 1228 cm^−1^ is present as a shoulder, revealing a large number of free ions for transport with the weakest ion pairing, which resulted in σ_25°C_ > 10^−2^ S cm^−1^ for both anions. The SRMs with *x* = 0 exhibited a minimal level of interaction between the SN matrix and salt. The mediators showed a minimal change in the positions of the modes for SN and salts. The change was noticed mainly at 769 cm^−1^ (762 cm^−1^; SN; δ_CH2_), 1228 cm^−1^ (1233 cm^−1^; SN; t_CH2_), 1443 cm^−1^ (1453 cm^−1^; ring), and 1474 cm^−1^ (1470 cm^−1^; ring) for both anions. The notations δ and t are the bending and twisting modes, respectively. The peak at 1228 cm^−1^ as a shoulder depicts the presence of ion pairs, too. The mediators therefore possessed lower σ_25°C_ values ≈ 10^−3^ S cm^−1^ for both anions. The SRMs with *x* = 0.5 showed a slightly higher level of interaction between the blend matrix and salt. The interaction was noticed through changes at 778 cm^−1^ (762 cm^−1^; Blend; δ_CH2_), 1472 cm^−1^ (1469 cm^−1^; Blend; δ_CH2_), 2253 cm^−1^ (2251 cm^−1^; Blend; ν_s,C≡N_), 2907 (TFSI^−^) or 2905 (Triflate^−^) cm^−1^ (2899 cm^−1^; Blend; ν_a,CH2_), and 2981 (TFSI^−^) or 2980 (Triflate^−^) cm^−1^ (2975 cm^−1^; Blend; ν_a,CH2_). In addition, the mediators depicted peaks at 1227 and 1334 cm^−1^, revealing ion pairing. Being uncharged and the same size as anions, coordinated ions have higher mobility in the polymeric matrix, resulting in more amorphous regions for ion transport [55,61]. This also reduces the number of free ions and the polymer matrix–salt interaction [55,61]. The anionic effect was observed in the ν_a,CH2_ mode only. The larger size of TFSI^−^ led to a higher blue shift in the ν_a,CH2_ mode, indicating a higher C−H bond contraction for forming more amorphous regions and, thereby, improving the σ_25°C_ value [32,34,35,62]. The SRMs with *x* = 1 portrayed the highest level of interaction between the PEO matrix and salt. This was due to the changes at 780 (TFSI^−^) or 777 (Triflate^−^) cm^−1^ (767 cm^−1^; ring), 1112 (TFSI^−^) or 1115 (Triflate^−^) cm^−1^ (1109 cm^−1^; PEO; ν_s,COC_), 1134 (TFSI^−^) or 1144sh (Triflate^−^) cm^−1^ (1149 cm^−1^; PEO; ν_CC_), 1349 (TFSI^−^) or 1343 (Triflate^−^) cm^−1^ (1342 cm^−1^; PEO; ω_s,CH2_), 2872 (TFSI^−^) or 2860 (Triflate^−^) cm^−1^ (2861 cm^−1^; PEO; ν_s,CH2_), and 2894 (TFSI^−^) or 2890 (Triflate^−^) cm^−1^ (2889 cm^−1^; PEO; ν_s,CH2_). These clearly show the anionic effect, where TFSI^−^ ions are more effective for C−H bond contraction and amorphous region formation, and therefore higher electrical conductivity. It is also noticeable that the ion pairing peaks neither were prominent nor appeared as shoulders; in fact, they overlapped with those of the PEO.

**Figure 4:**
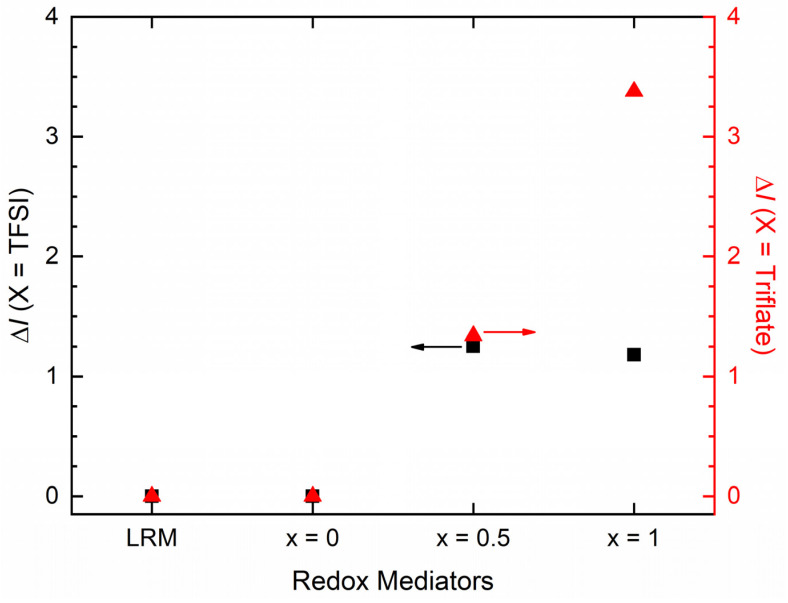
Relative intensity, Δ*I*, of SRM [(1 − *x*)SN: *x*PEO]-LiX-Co salts with *x* = 0, 0.5, and 1, and X = TFSI^−^ and Triflate^−^, along with those of their liquid counterparts (LRMs).

The level of interaction between the matrix and salt in the redox mediators is observed in the following order: LRM < *x* = 0 < 0.5 < 1. This order is the same for the anionic effect, too. We quantified the matrix–salt interaction by evaluating the relative intensity, $∆I=I_{1105}/I_{1196},$ for both anions of redox mediators, where $I_{1105}$ stands for intensity at 1105 cm^−1^ for the ν_s,COC_ mode of PEO, and $I_{1196}$ for the strongest peak at 1196 cm^−1^ for the ν_a,CF3_ mode of ionic salts. Figure 4 shows the relative intensity of LRMs and SRMs for both anions. The LRMs and SRMs (*x* = 0) possessed ΔI = 0 for both anions, indicating the least interaction between the solvent or matrix and ionic salt. The value of ΔI(X = TFSI^−^) was ≈ 1.2 for the SRMs with *x* = 0.5 and 1, while the PEO-based SRMs with *x* = 0.5 and 1 had a higher value of ΔI(X = Triflate^−^): 1.34 and 3.4, respectively. This revealed an increase in the level of interaction from *x* = 0.5 to 1, resulting in a hindrance in ion transport and a decrease in σ_25°C_ value. This also demonstrated that ΔI(X = Triflate^−^) is greater than ΔI(X = TFSI^−^), indicating the anionic effect. The XRD study, discussed below, also supports this result. 

**Figure 5:**
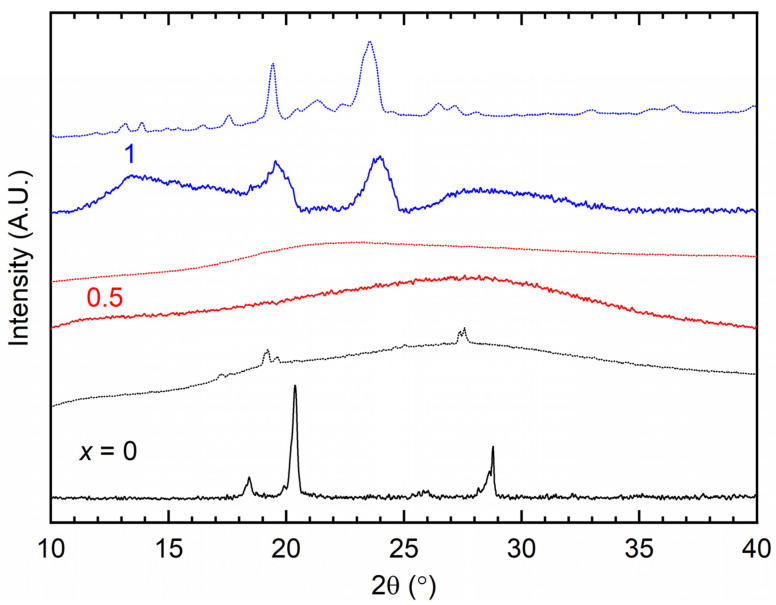
XRD patterns of SRM [(1 − *x*)SN: *x*PEO]-LiX-Co salts, where *x* = 0, 0.5, and 1. X = TFSI^−^ (solid line) and Triflate^−^ (dotted line).

Figure 5 shows the XRD patterns of the SRM [(1 − *x*)SN: *x*PEO]-LiX-Co salts with *x* = 0, 0.5, and 1 for X = TFSI^−^ (solid line) and Triflate^−^ (dotted line). Table 2 lists the observed peaks of redox mediators with *x* = 0 and 1, which are small and broad as well as slightly shifted relative to the matrix (SN: 2θ ≈ 19.7° and 28.1°; PEO: 2θ ≈ 19.2° and 23.3° [32]). The patterns of these mediators had no peaks for other ingredients. In contrast, the redox mediators with *x* = 0.5 exhibited no peaks. An amorphous peak appeared at 27.6° for X = TFSI^−^ and 23.1° for X = Triflate^−^. These indicate disorder in the SN molecules, eutectic phase formation, and amorphous phase formation in the PEO or blend matrix [3,21,33,34,35,63]. For the blend-based redox mediators (*x* = 0.5), PEO acted as an impurity, abolishing the crystalline structure of the SN in the presence of ions [3,33,34,35]. The absence of reflection peaks of the ionic salts also confirmed complete dissolution and complexation of the salts. The anionic effect is visible for all the redox mediators, *x* = 0 − 1. The redox mediator with *x* = 0 exhibited relatively stronger reflection peaks of succinonitrile for X = TFSI^−^ than Triflate^−^. The latter is indicative of eutectic phase formation, as indicated by the DSC study, which is discussed later [21,63]. The redox mediator (*x* = 1) had stronger reflection peaks of PEO for X = Triflate^−^ than TFSI^−^, revealing a higher level of PEO crystallinity and, thereby, a lower value of electrical conductivity. The SEM results, discussed later, corroborate these results.

**Figure 6:**
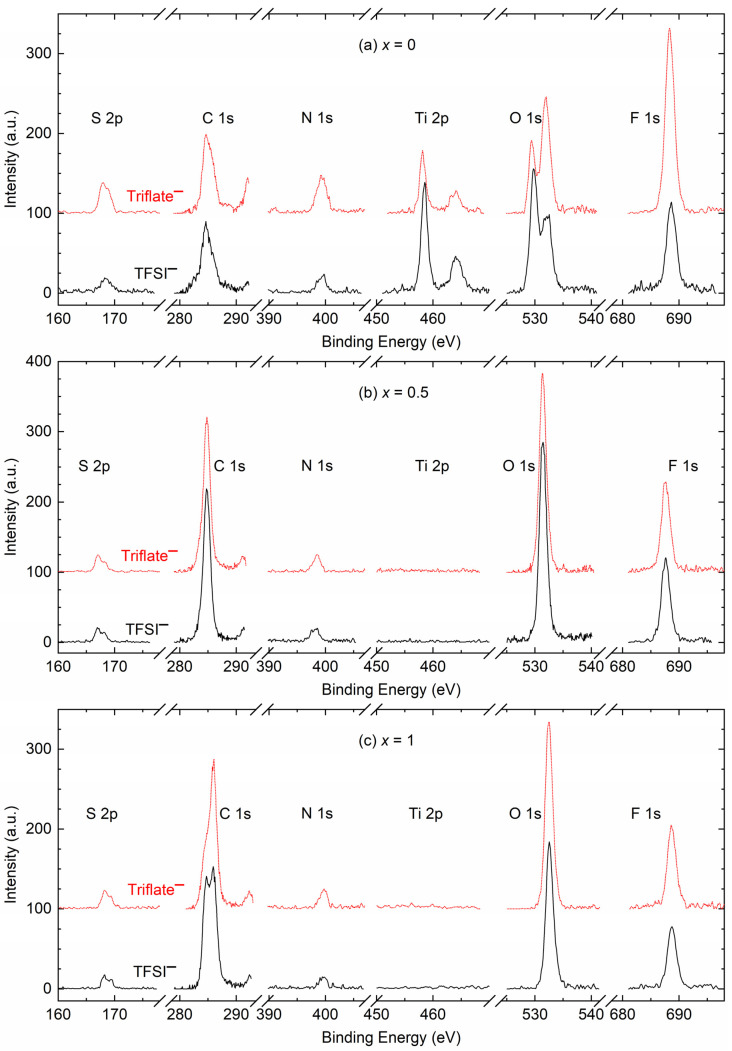
XPS spectra of different elements of SRM [(1 − *x*)SN: *x*PEO]-LiX-Co salts, where *x* = (**a**) 0, (**b**) 0.5, and (**c**) 1. X = TFSI^−^ (solid line) and Triflate^−^ (dotted line).

**Figure 7:**
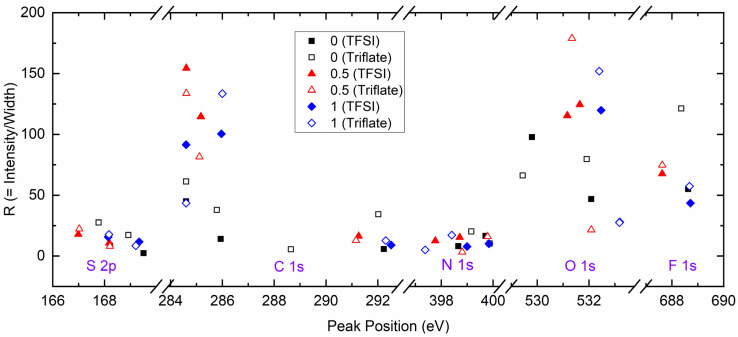
Plot of ratio (R) of intensity with width vs. peak position for different elements of SRM [(1 − *x*)SN: *x*PEO]-LiX-Co salts, where *x* = 0, 0.5, and 1. X = TFSI^−^ (solid symbols) and Triflate^−^ (open symbols).

X-ray photoelectron spectroscopy is a tool used to study the interaction between ingredients at the surface [47,64,65,66,67,68]. Figure S2 shows the survey spectra of SRM [(1 − *x*)SN: *x*PEO]-LiX-Co salts (*x* = 0, 0.5, and 1; X = TFSI^−^ and Triflate^−^). The survey spectrum was corrected to fix the C 1 s peak at 284.6 eV [64,65,66]. The survey spectra depicted S 2p, C 1 s, N 1 s, O 1 s, and F 1 s elements for all redox mediators. However, only the redox mediators with *x* = 0 exhibited Ti 2p peaks. The presence of this peak can be attributed to the enhanced penetration of SN-based electrolytes into the pores of the TiO_2_ layer and the creation of a remarkably thin film on TiO_2_, facilitated by the solid solvent characteristic of SN. The SEM image demonstrates the infiltration and development of a thin coating of SN-based electrolyte on the TiO_2_ substrate, as described in the next paragraph. The TiO_2_ substrate is anticipated to exhibit a substantial accumulation of PEO-based redox mediators (*x* = 0.5 and 1), hence impeding the examination of the electrolyte/TiO_2_ interface. The peak’s position, intensity, and width (full width at half maximum) were determined through the fitting of the smoothed and baseline-corrected spectrum. Figures S3–S5 depict the best-fit plots of different elements of SRMs with *x* = 0, 0.5, and 1, respectively. The left and right columns are for SRMs, with X = TFSI^−^ and Triflate^−^, respectively. Figure 6 shows smoothed and baseline-corrected XPS spectra of the S 2p, C 1 s, N 1 s, O 1 s, and F 1 s elements for the SRM [(1 − *x*)SN: *x*PEO]-LiX-Co salts with *x* = (a) 0, (b) 0.5, and (c) 1, where X = TFSI^−^ and Triflate^−^. Figure 6a also portrays the spectra of the Ti 2p element. The peaks can be assigned in light of previously reported studies [47,64,65,66,67,68]. The SRMs depicted a small-height S 2p peak because of the −SO_2_^−^ group at ≈168.2 eV for the spin of 3/2, associated with a shoulder peak at ≈169.8 eV for the spin of ½. The area of the C 1 s core level is the most complex. This area showed a standard mid-height peak at 284.6 eV for the alkyl (−C−C−) group associated with a strong peak at ≈286 eV for the bpy ring or −C−C−O− group, a small-height peak at ≈288.6 eV for the −C≡N group, and a distinctive small-height peak at ≈292 eV for the –CF_3_ group. The N 1 s spectrum depicted a small-height broad peak consisting of two deconvoluted peaks because of anions at ≈398 eV and the bpy ring at ≈400 eV. The Ti 2p spectrum of the TiO_2_ layer exhibited two strong and distinctive peaks at ≈458.2 eV and ≈464 eV because of the spin-orbit-coupling phenomenon. The SRMs with *x* = 0 portrayed two O 1 s core-level strong distinctive peaks at ≈529.8 eV and ≈532 eV because of the −SO_2_^−^ group in the anions. The PEO-based SRMs (*x* = 0.5 and 1) exhibited the strongest O 1 s peak with deconvolution of ≈531 eV and ≈532 eV because of the spin-orbit-coupling phenomenon. The redox mediators also showed a strong F 1 s core-level distinctive peak at ≈688.6 eV because of the –CF_3_ group of anions. We divided the intensity by the width to make the ratio dimensionless. Figure 7 plots the intensity/width (=R) against the peak position for all redox mediators with *x* = 0, 0.5, and 1. The solid symbols represent redox mediators with X = TFSI^−^, and open symbols denote redox mediators with X = Triflate^−^. This figure demonstrates a shift either in the ratio (Δ*R* = *R*_TFSI_ − *R*_Triflate_; cf. Figure S6) or peak position (Δ*P* = Peak Position_TFSI_ − Peak Position_Triflate_; cf. Figure S7) or both, revealing an anionic effect. The level of shift differed depending on the composition and element. For example, the SRMs with *x* = 0 showed Δ*R* < 0, except for O 1 s, where Δ*R* > 0, too; the redox mediators with *x* = 0.5 depicted Δ*R* > 0, except for O 1 s and F 1 s, where Δ*R* < 0; and the redox mediators with *x* = 1 exhibited both positive and negative Δ*R*. At F 1 s, the negative shift was minimum for *x* = 0.5 and maximum for *x* = 0. The SRMs with *x* = 0 showed Δ*P* > 0 for S 2p, C 1 s, O 1 s, and F 1 s core levels, and Δ*P* < 0 for N 1 s. The redox mediators with *x* = 0.5 exhibited Δ*P* ≥ 0 for S 2p, C 1 s, and F 1 s core levels, and Δ*P* < 0 for N 1 s and O 1 s. The redox mediators with *x* = 1 depicted Δ*P* ≥ 0 for all core levels. 

**Figure 8:**
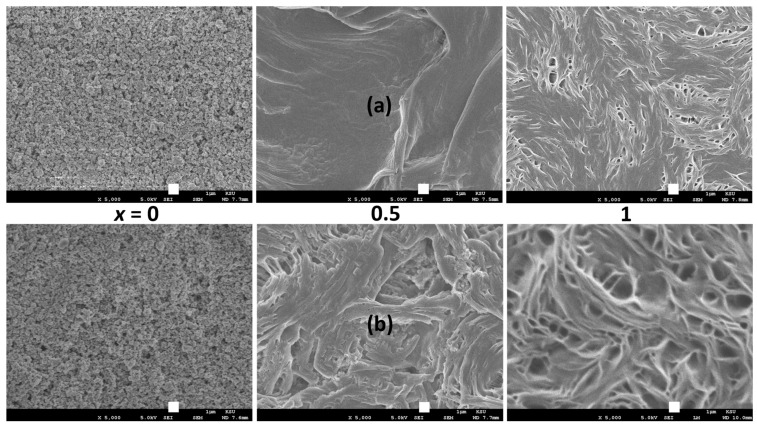
SEM images for the surface of SRM [(1 − *x*)SN: *x*PEO]-LiX-Co salts with *x* = 0, 0.5, and 1, infiltrated in the mesoporous TiO_2_. X = (**a**) TFSI^−^ and (**b**) Triflate^−^. Scale bar, 1 μm.

The mesoporous TiO_2_ layer was subjected to impregnation with a solid redox mediator, [(1 − *x*)SN: *x*PEO]-LiX-Co salts, with *x* = 0, 0.5, and 1, and X = TFSI^−^ or Triflate^−^. Figure 8 shows SEM images of the surface of the impregnated mesoporous TiO_2_. The pores of mesoporous TiO_2_ were penetrated by redox mediators with *x* = 0 due to the solid solvent feature of SN. On the other hand, it was observed that the redox mediators with *x* = 0.5 and 1 failed to effectively impregnate the pores of the mesoporous TiO_2_, as evidenced by the subpar photovoltaic performance [69]. PEO-containing redox mediators (*x* = 0.5 and 1) exhibited fibril-like structures due to the presence of polymeric chains of PEO [10,34,35]. Succinonitrile, as a solid solvent and plasticizer, exerted significant influence over the fibril architectures [34,35]. A similar trend may be observed in the case of SRMs, where the surface smoothness exhibited a reduction from *x* = 0.5 to 1, as well as from X = TFSI^−^ to Triflate^−^. These findings suggest a rise in the crystallinity of PEO within the range of *x* = 0.5 to 1, as well as from X = TFSI^−^ to Triflate^−^. This observation is supported by the transmittance spectra of the redox mediators (*x* = 0, 0.5, and 1; X = TFSI^−^ to Triflate^−^), which are described below.

**Figure 9:**
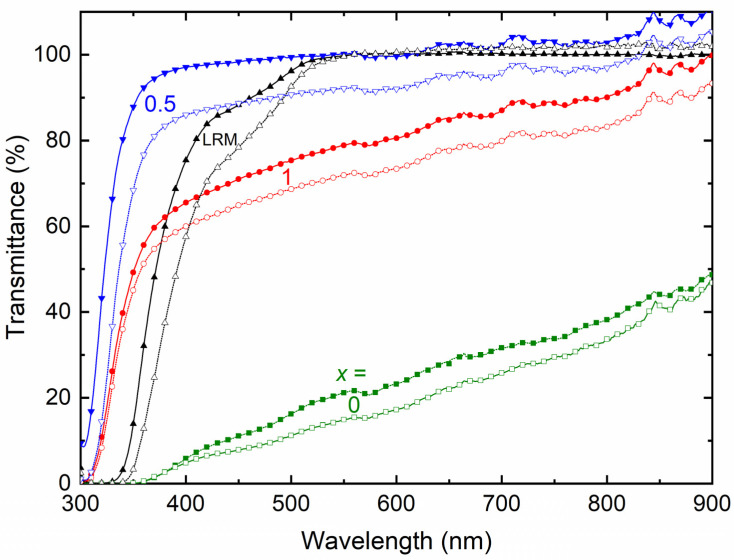
Transmittance spectra of SRM [(1 − *x*)SN: *x*PEO]-LiX-Co salts with *x* = 0, 0.5, and 1. LRM, ACN-based liquid counterpart. X = TFSI^−^ (filled symbols) and Triflate^−^ (open symbols).

Figure 9 shows the transmittance spectra of SRM [(1 − *x*)SN: *x*PEO]-LiX-Co salts (*x* = 0, 0.5, and 1) for X = TFSI^−^ and Triflate^−^. We can divide the spectra into three regions: I (UV-A; 350 nm), II (visible; 555 nm), and III (near-IR; 900 nm). Table 2 summarizes the transmittance of the SRMs, which can be compared with those of their liquid counterparts (LRMs): 13.9% (I), 99.9% (II), and 99.7% (III) for X = TFSI^−^, and 3.3% (I), 99.9% (II), and 101.9% (III) for X = Triflate^−^. The SRMs with *x* = 0 and 1 had poor transmittance values; however, blending SN and PEO largely improved the transmittance. This is due to a decrease in PEO crystallinity because of the plasticizing properties of the SN [35]. Table 2 portrays the anionic effect, too, where TFSI^−^ led to better transmittance than Triflate^−^. This is due to a decrease in PEO crystallinity, as pointed out by the DSC results, which are discussed below. 

**Figure 10:**
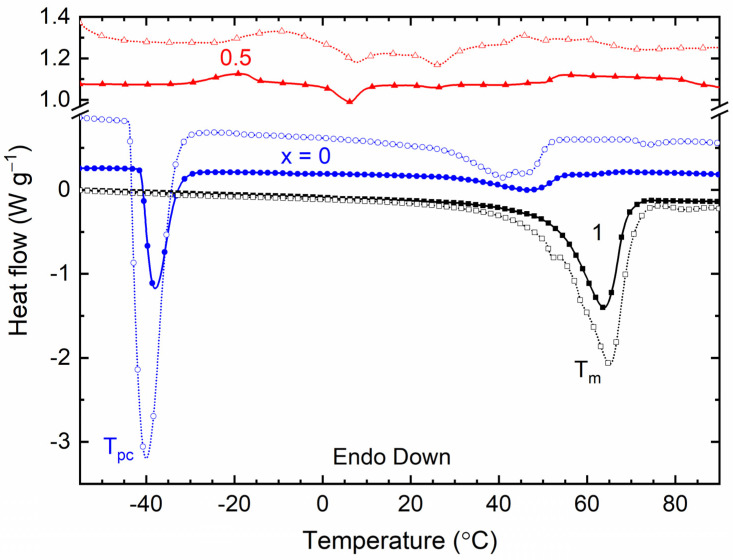
DSC curves of SRM [(1 − *x*)SN: *x*PEO]-LiX- Co salts with *x* = 0, 0.5, and 1. X = TFSI^−^ (filled symbols) and Triflate^−^ (open symbols).

Figure 10 shows the DSC curves of the SRM [(1 − *x*)SN: *x*PEO]-LiX-Co salts, where *x* = 0, 0.5, and 1, and X = TFSI^−^ and Triflate^−^. The SRMs with *x* = 0 portrayed two endothermic peaks marked by *T*_pc_ and *T*_m_ for crystal-to-plastic-crystal phase transition temperature and melting temperature, respectively. Table 3 lists the values of *T*_pc_ and *T*_m_ for comparison. For the SN matrix, *T*_pc_ = –38.4 °C and *T*_m_ = 57.7 °C [21,63]. It is worth mentioning that the area of *T*_m_-peak corresponds to the heat enthalpy or crystallinity of the redox mediator [21,32]. We observed that the position and area of *T*_m_-peak decreased for the SRMs with *x* = 0 relative to the pure SN matrix. This is indicative of a decrease in the crystallinity of succinonitrile [21]. The *T*_pc_-peak showed a position similar to that of the pure matrix, as observed earlier for the SN-LiI-I_2_ redox mediator, however, with an increase in the area, most probably because of the SN–ionic salt interaction [21,63]. The anionic effect is also noticeable for SRMs with *x* = 0. For example, TFSI^−^ resulted in a broad *T*_m_-peak, which is indicative of the disordered plastic crystalline nature of SN. In contrast, Triflate^−^ yielded multiplets at higher temperatures with a larger area, indicating eutectic phase formation along with the disordered plastic crystal phase of SN. TFSI^−^ also resulted in the area (40.9) of the *T*_pc_-peak being less than that for Triflate^−^ (139.9), which is indicative of less crystallinity and, thereby, higher electrical conductivity in the TFSI^−^-based redox mediator. The SRMs with *x* = 1 had a *T*_m_-peak with values of only 63.8 °C for X = TFSI^−^ and 65.2 °C for X = Triflate^−^, which are less than the *T*_m_-value of the PEO matrix (65.7 °C [32]). The area of the *T*_m_-peak was also less than that of the PEO matrix, revealing a decrease in PEO crystallinity. These DSC curves also exhibited the anionic effect via a decrease in the position and area of the *T*_m_-peak for X = TFSI^−^, revealing a decrease in PEO crystallinity and, thereby, an increase in electrical conductivity [32,33,34,35]. The SRMs with *x* = 0.5 possessed negligibly small and broad *T*_m_-peaks with values of only 4 °C for X = TFSI^−^ and 7.8–26 °C for X = Triflate^−^. These values, as well as the peak area, are less than those of the PEO-SN blend matrix (*T*_m_ ≈ 30.1 °C [32]), indicating a decrease in PEO crystallinity. The anionic effect was visualized at the *T*_m_-peak position and area. TFSI^−^ led to lower values of *T*_m_ and a smaller area compared to Triflate^−^, resulting in lower PEO crystallinity and, thereby, a higher σ_25°C_ value. These results are also consistent with the findings of the TGA investigation, which are discussed below. 

**Figure 11:**
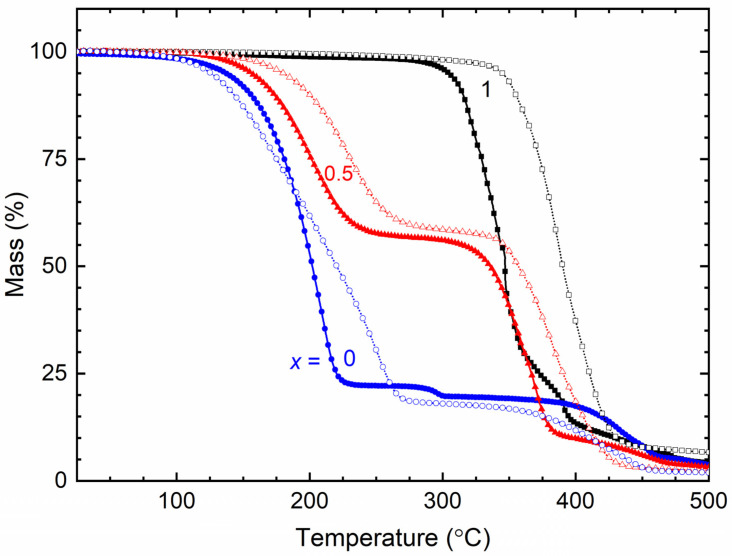
TGA curves of SRM [(1 − *x*)SN: *x*PEO]-LiX-Co salts with *x* = 0, 0.5, and 1. X = TFSI^−^ ions (filled symbols) and Triflate^−^ ions (open symbols).

Figure 11 shows the TGA curves of the SRM [(1 − *x*)SN: *x*PEO]-LiX-Co salts with *x* = 0, 0.5, and 1 for X = TFSI^−^ and Triflate^−^. The initial plateau region of the curve corresponds to the thermal stability of the SRM, which is ≈75 °C for *x* = 0, 125 °C for *x* = 0.5, and 200 °C for *x* = 1. These values are similar to those of the pure matrices [32]. The SRMs with *x* = 0 and 1 exhibited one-step dropping due to the decomposition of the matrix, while the mediators with *x* = 0.5 showed two-step dropping due to the decomposition of the SN and PEO matrices [3,32]. These curves also portrayed an anionic effect via the degradation phenomenon. TFSI^−^ resulted in more rapid degradation compared to Triflate^−^, particularly for SRMs with *x* = 0.5 and 1. This is because TFSI^−^-based redox mediators have lower PEO crystallinity.

As mentioned earlier, an SRM with σ_25°C_ > 10^−4^ S cm^−1^ and *E*_a_ < 0.3 eV is required to regenerate dye molecules faster at the TiO_2_/dye/electrolyte interface and, thereby, inhibit back-electron transfer [6,7,8,9,10,11,12,13,14,15,16,17,18,19]. Faster dye regeneration via the faster oxidation of ionic species results in a higher photocurrent and cell efficiency. This also results in faster ionic species regeneration. However, the higher mass of metal ions, e.g., Co^2+^/Co^3+^, slows down their diffusion in DSSCs, which decreases the concentration of metal ions at the TiO_2_/dye/electrolyte interface, resulting in poor cell efficiency [69,70]. Hence, it is imperative to enhance the porosity of the mesoporous TiO_2_ film in order to accommodate a greater number of ionic species at the interface [70]. It is also necessary to have TiO_2_ pores that are water-free because water changes the surface of TiO_2_ and decreases cell efficiency [4,71]. In the present scenario, due to their superior electrical transport and optical properties, the DSSCs with TFSI^−^-based SRMs are expected to perform better than those with Triflate^−^-based SRMs. However, porosity at the TiO_2_/dye/electrolyte interface needs to be optimized by utilizing a mixture of large and small anatase TiO_2_ nanoparticles [69,70,72,73,74]. Therefore, we will perform DSSC fabrication and characterizations in the future. 

## 4. Conclusions

We studied the electrical transport properties of SRM [(1 − *x*)SN: *x*PEO]-LiX-Co salts (*x* = 0, 0.5, and 1; X = TFSI^−^ and Triflate^−^), and the results were compared with those of their acetonitrile-based liquid counterparts (LRMs). The LRMs exhibited σ_25°C_ ≈ 10^−2^ S cm^−1^. The SRMs achieved σ_25°C_ ≈ 10^−3^ S cm^−1^ for *x* = 0, >10^−4^ S cm^−1^ for *x* = 0.5, and ≈10^−6^ S cm^−1^ for *x* = 1. In these redox mediators, σ_25°C_(TFSI^−^) > σ_25°C_(Triflate^−^) and *E*_a_(TFSI^−^) < *E*_a_(Triflate^−^). FT-IR spectroscopy showed the interaction and, thereby, the anionic effect in the following order: LRM < 0 < 0.5 < 1. The XRD study exhibited an increase in PEO crystallinity from *x* = 0.5 to 1, which was more for Triflate^−^ ions. The XPS study showed a shift in the intensity/width ratio and peak position of the elements because of the anionic effect. The SEM images depicted an increase in surface roughness from *x* = 0.5 to 1, which was higher for Triflate^−^ ions. The transmittance showed the following orders: LRM = 0 (= 0%) << 1 << 0.5 in the UV-A region, 0 << 1 << LRM ≈ 0.5 in the visible region, and 0 << 1 < 0.5 < LRM in the near-IR region. Only the SRMs with *x* = 0.5 showed higher transmittance in the UV-A, visible, and near-IR regions. In addition, the transmittance was higher for the TFSI^−^ ion-based redox mediators. The DSC study exhibited the lowest *T*_m_-peak area for *x* = 0.5 and the highest peak area for *x* = 1. The SRMs had a lower peak area for TFSI^−^ ions. The TGA study revealed the thermal stability of SRMs, ≈75 °C for *x* = 0, 125 °C for *x* = 0.5, and 200 °C for *x* = 1, and degradation was faster for TFSI^−^ ion-based redox mediators. Various studies on [(1 − *x*)SN: *x*PEO]-LiX-Co salts (*x* = 0, 0.5, and 1) have demonstrated similar trends despite different anions (X = TFSI^−^ and Triflate^−^). However, the electrical transport properties of SRMs with X = TFSI^−^ were better than those of SRMs with X = Triflate^−^. Owing to superior its electrical transport and optical properties, the SRM with *x* = 0.5 and X = TFSI^−^ can be utilized for DSSC and tandem solar cell applications.

## Figures and Tables

**Table 1:** Electrical transport properties of SRM [(1 − x)SN: *x*PEO]-LiX-Co salts (*x* = 0, 0.5, and 1; X = TFSI^−^ or Triflate^−^). LRM, ACN-based liquid counterpart.

*x* (Wt. Fraction)	σ_25°C_ (S cm^−1^)		Log σ − *T*^−1^ Nature	Activation Energy (eV) in Regions I and II	
	TFSI^−^	Triflate^−^		TFSI^−^	Triflate^−^
0	2.1 × 10^−3^	1.5 × 10^−3^	Arrhenius	0.56 (I), 0.16 (II)	0.77 (I), 0.13 (II)
0.5	7.2 × 10^−4^	3.1 × 10^−4^	VTF	0.05	0.06
1	9.7 × 10^−7^	6.3 × 10^−7^	Arrhenius	1.07 (I), 0.36 (II)	1.22 (I), 0.44 (II)
LRM	1.7 × 10^−2^	1.6 × 10^−2^	Arrhenius	0.15	0.16

**Table 2:** Angle (2θ) and transmittance (T) of SRM [(1 − *x*)SN: *x*PEO]-LiX-Co salts, where *x* = 0, 0.5, and 1. X = TFSI^−^ and Triflate^−^. I, II, and III stand for UV-A, visible, and near-IR regions, respectively.

*x* (Wt. Fraction)	2θ (°)		T (%) in Regions I, II, and III	
	TFSI^−^	Triflate^−^	TFSI^−^	Triflate^−^
0	20.4, 28.8	19.2, 27.6	0 (I), 21.6 (II), 48.7 (III)	0 (I), 15.1 (II), 46.8 (III)
0.5	27.6	23.1	88.1 (I), 99.8 (II), 110.5 (III)	68.8 (I), 91.8 (II), 105.4 (III)
1	19.5, 24	19.4, 23.6	49.6 (I), 78.9 (II), 99.9 (III)	45.4 (I), 71.9 (II), 93.3 (III)

**Table 3:** *T*_m_, *T*_m_-area, and *T*_pc_ of SRM [(1 − *x*)SN: *x*PEO]-LiX-Co salts with *x* = 0, 0.5, and 1. X = TFSI^−^ and Triflate^−^.

*x* (Wt. Fraction)	*T*_m_ (°C)		*T*_m_-Area (a.u.)		*T*_pc_ (°C)	
	TFSI^−^	Triflate^−^	TFSI^−^	Triflate^−^	TFSI^−^	Triflate^−^
0	47	41 and 45.8	15.6	43.2	−37.8	−40
0.5	4	7.8 and 26	2.4	17.5	-	-
1	63.8	65.2	92.7	160.4	-	-

## Data Availability

The data are contained within the article.

## Supplementary Materials

The following supporting information can be downloaded at: https://www.mdpi.com/article/10.3390/polym16101436/s1, Figure S1: Chemical structure of (a) ionic salts and (b) matrices; Figure S2. XPS survey spectra of solid redox mediators, [(1 − *x*)SN: *x*PEO]-LiX-Co salts (*x* = 0, 0.5, and 1; X = TFSI^−^ and Triflate^−^); Figure S3: Best fits of XPS spectra of different elements of solid redox mediators (*x* = 0; X = TFSI^−^ and Triflate^−^). This figure also includes the best fits of XPS spectra of the Ti 2p element; Figure S4: Best fits of XPS spectra of different elements of solid redox mediators (*x* = 0.5; X = TFSI^−^ and Triflate^−^); Figure S5: Best fits of XPS spectra of different elements of solid redox mediators (*x* = 1; X = TFSI^−^ and Triflate^−^); Figure S6: A plot of ΔR (= [intensity/width]_TFSI_ − [intensity/width]_Triflate_) with peak position for different elements of solid redox mediators, [(1 − *x*)SN: *x*PEO]-LiX-Co salts, where *x* = 0, 0.5, and 1. X = TFSI^−^ and Triflate^−^; Figure S7: A plot of ΔP (= [peak position]_TFSI_ − [peak position]_Triflate_) with peak position for different elements of solid redox mediators, [(1 − *x*)SN: *x*PEO]-LiX-Co salts, where *x* = 0, 0.5, and 1. X = TFSI^−^ and Triflate^−^; Table S1: Abbreviations used in the article; Table S2: Chemicals used for the preparation of liquid and solid redox mediators; Table S3: Details of characterization techniques; Table S4: Equipment used for the measurements.
