# Supplementary material for: Anionic Effect on Electrical Transport Properties of Solid Co2+/3+ Redox Mediators
Source: Polymers (Basel). 2024 May 19;16(10):1436. doi: 10.3390/polym16101436 (PMC11124796; doi:10.3390/polym16101436)
Supplement: Supplementary file 1 [file polymers-16-01436-s001.zip › polymers-2695123-supplementary.pdf]

Anionic Effect on Electrical Transport Properties of Solid $Co^{2+/3+}$ Redox Mediators

Ravindra Kumar Gupta *, Ahamad Imran and Aslam Khan

King Abdullah Institute for Nanotechnology, King Saud University, Riyadh 11451, Saudi Arabia
* Correspondence: rgupta@ksu.edu.sa

Supplementary Information

Table S1. Abbreviations used in the article.

ACN, acetonitrile
SN, succinonitrile
PEO, poly(ethylene oxide)
bpy, tris-(2,2'-bipyridine)
TFSI, bis(trifluoromethyl) sulfonylimide
Triflate, trifluoromethanesulfonate
DSSC, dye-sensitized solar cell
SRM, solid redox mediator
LRM, liquid redox mediator
IS, impedance spectroscopy
FT-IR, Fourier-transform infrared spectroscopy
XRD, X-ray diffractometry
XPS, X-ray photoelectron spectroscopy
SEM, scanning electron microscopy
UV-vis, UV-visible spectroscopy
DSC, differential scanning calorimetry
TGA, thermogravimetric analysis
σ, electrical conductivity
R_b, bulk resistance
R_s, series resistance due to leads
C_1, chemical capacitance
C_2, double-layer capacitance
E_a, activation energy
σ_o, pre-exponential factor
k_B, Boltzmann constant
B, pseudo-activation energy
T_o, free-volume temperature
T_m, melting temperature
T_{pc}, crystal-to-plastic-crystal phase transition temperature
I_{1105}, intensity at 1105 cm^{-1} for the $\nu_{s,COC}$ mode of PEO
I_{1196}, intensity at 1196 cm^{-1} for the $\nu_{a,CF3}$ mode of ionic salts
$\Delta I = I_{1105}/I_{1196}$
R = intensity/width
$\Delta R = R_{TFSI} - R_{Triflate}$
ΔP = Peak Position$_{TFSI}$ − Peak Position$_{Triflate}$

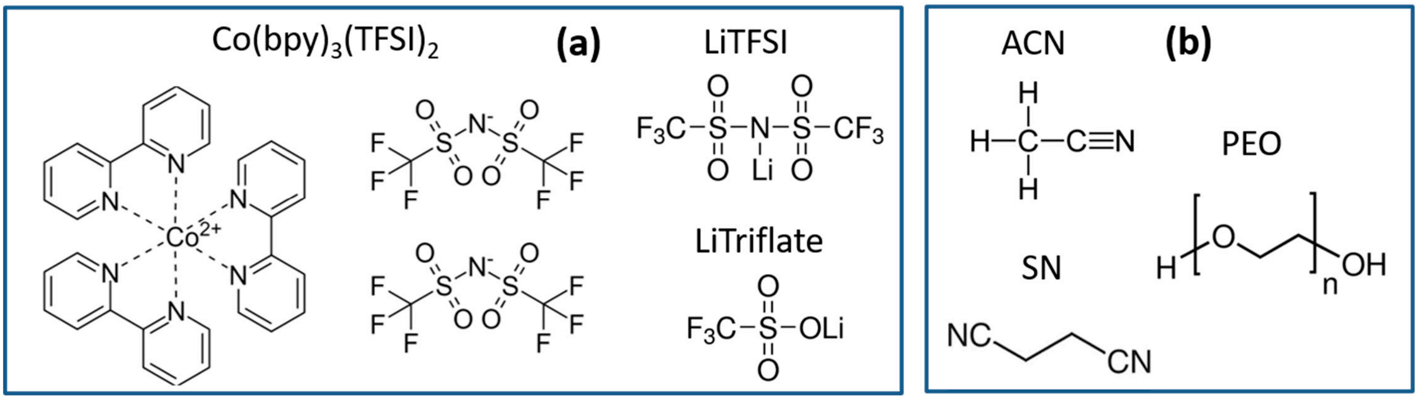

Figure S1. Chemical structure of (a) ionic salts and (b) matrices.

Table S2. Chemicals used for the preparation of liquid and solid redox mediators.

Chemicals	Purity (%)	Make
**Acetonitrile (ACN)**	99.9	Fisher Scientific, USA
**Succinonitrile**	99	Sigma-Aldrich, Inc., USA
**Poly(ethylene oxide)**	99	Sigma-Aldrich, Inc., USA
**$Li(CF_3SO_2)_2N$**	99.9	Sigma-Aldrich, Inc., USA
**$LiCF_3SO_3$**	96	Sigma-Aldrich, Inc., USA
**$Co(bpy)_3(TFSI)_2$**	-	Dyenamo, Sweden
**$Co(bpy)_3(TFSI)_3$**	-	Dyenamo, Sweden

Table S3. Details of characterization techniques.

Technique	Details
**IS**	We poured a liquid electrolyte (LRM or SRM with x = 0 in liquid form) into the space of a sample holder [1]. This space with an area (A) of 0.16 cm^2 and a thickness (l) of 0.05 cm was produced by a Teflon spacer between platinum plates (the blocking electrode). We placed a thick film of PEO-based SRMs (x = 0.5 and 1) between the stainless steel plates (blocking electrode; diameter = 1 cm) of another sample holder [2]. The electrolyte was subjected to a 20 mV AC voltage with a frequency range of 10^5-1 Hz using an impedance analyzer. The resulting Nyquist curve helped to deduce the bulk resistance and, thereby, the electrical conductivity. The temperature-dependent measurement of electrical conductivity helped to determine the activation energy.
**FT-IR**	We formed a thin film of electrolyte on a KBr pellet (diameter 2.5 cm; thickness 0.2 cm). We collected the FT-IR spectrum of this film from 4000 to 400 cm^{-1} with a resolution of 1 cm^{-1} at 25 °C using an FT-IR spectrometer.
**XRD**	A thick film of electrolyte was formed on a cover glass (area 1 cm^2). This film was subjected to CuKα radiation (1.54184 Å) in a range of 10–40° with a step of 0.06° using a diffractometer. The amorphous profile was smoothed to decrease the noise-to-signal ratio.
**XPS**	A mesoporous TiO_2 (size 18 nm) layer with an area of 1 cm^2 and thickness of 7 μm on a microscopic glass was coated with the electrolyte. We subjected this film to MgKα X-ray radiation (1253.6 eV) under ultra-high vacuum conditions using an XPS unit. We scanned the film five times, from 1000 to 0 eV, using a step of 1 eV to survey the elements. We precisely determined the elements' position by scanning ten times at a step of 0.1 eV for 100 ms. The spectra of elements were corrected to fix the C 1s peak at 284.6 eV, smoothed for 5 points, and finally corrected for the rubberband baseline. We used software to perform the best fit and deconvolute the peak. The intensity of the peak was divided by the width (full width at half maxima) to make the ratio dimensionless.
**SEM**	A film of electrolyte was formed on a mesoporous TiO_2 layer (thickness 7 μm) on a microscopic glass (area 1 cm^2). This glass was broken into small pieces. A very small piece of this film was coated with platinum using a JEOL DC sputtering unit, and an image was obtained using a JEOL scanning electron microscope.
**UV-vis**	We formed a micron-thick electrolyte film on a cover glass (area 1.5 cm^2). We recorded the transmittance spectrum of the film using a UV-visible spectrometer.
**DSC**	We sealed an aluminum crucible containing nearly 10 mg of electrolyte. This crucible was subjected to heat flow measurement from –50 °C to 90 °C at 10 °C per minute using a DSC unit in the N_2 gas environment.
**TGA**	We covered an alumina pan containing nearly 10 mg of electrolyte and used a TGA unit in a nitrogen gas environment to measure the weight loss from room temperature to 600 °C at 10 °C per minute.

Table S4. Equipment used for the measurements.

Technique	Equipment
**IS**	Model PalmSens4, Palmsens impedance analyzer, Houten, the Netherlands
**FT-IR**	Model Spectrum 100, Perkin Elmer FT-IR spectrometer, Waltham, USA
**XRD**	Model D2 Phaser, Bruker X-ray diffractometer, Karlsruhe, Germany
**XPS**	Model JPS-9030, JEOL X-ray photoelectron spectrometer, Tokyo, Japan
**SEM**	Model JSM-7600F, JEOL scanning electron microscope, Tokyo, Japan
**UV-vis**	Model 8453, Agilent UV-visible spectrometer, Santa Clara, USA
**DSC**	Model DSC-60A, Shimadzu differential scanning calorimeter, Kyoto, Japan
**TGA**	Model DTG-60H, Shimadzu TGA unit, Kyoto, Japan

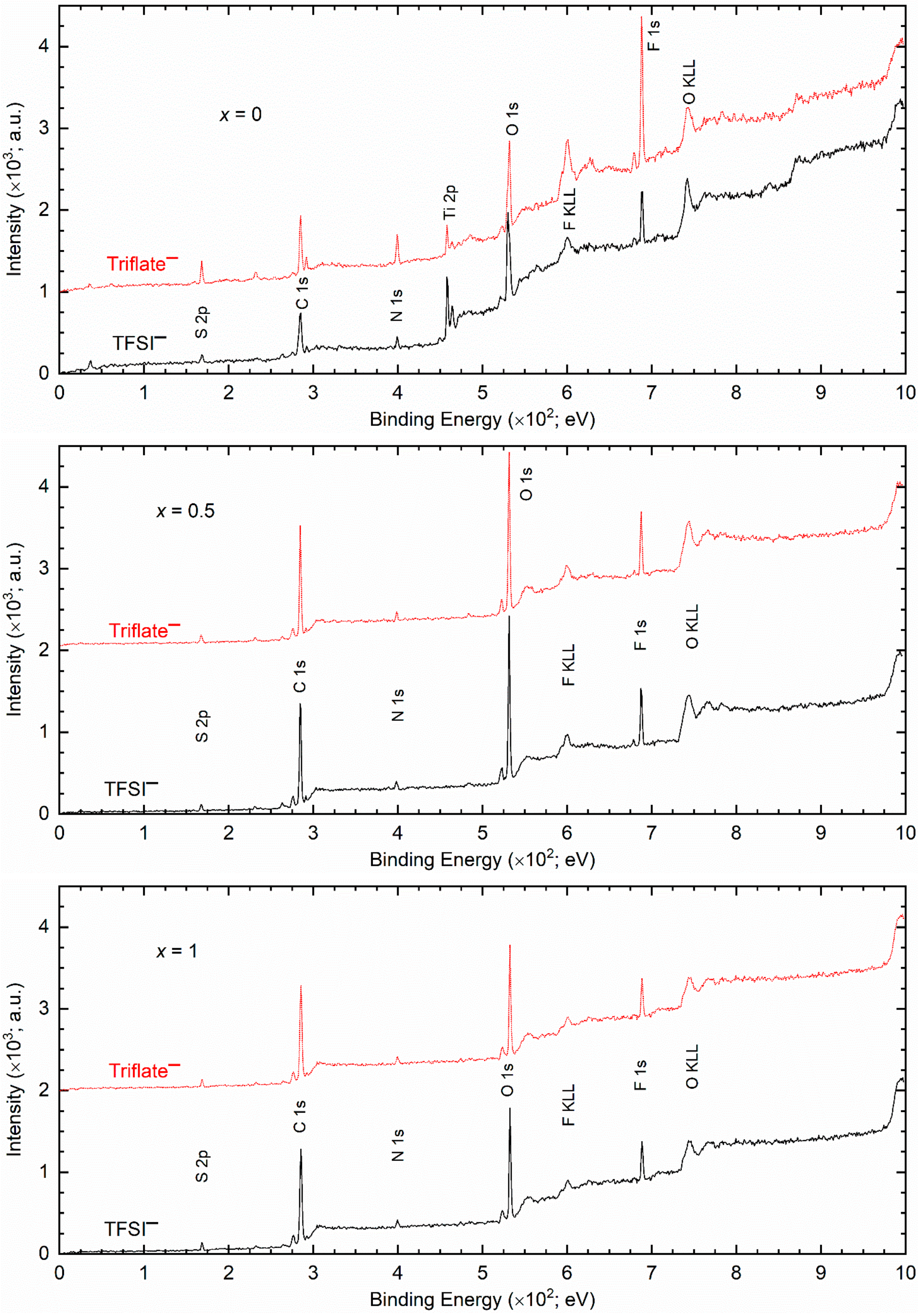

Figure S2. XPS survey spectra of solid redox mediators, [(1–x)SN: xPEO]-LiX-Co salts (x = 0, 0.5, and 1; X = TFSI⁻ and Triflate⁻).

$x = 0$

X = TFSI⁻	X = Triflate⁻
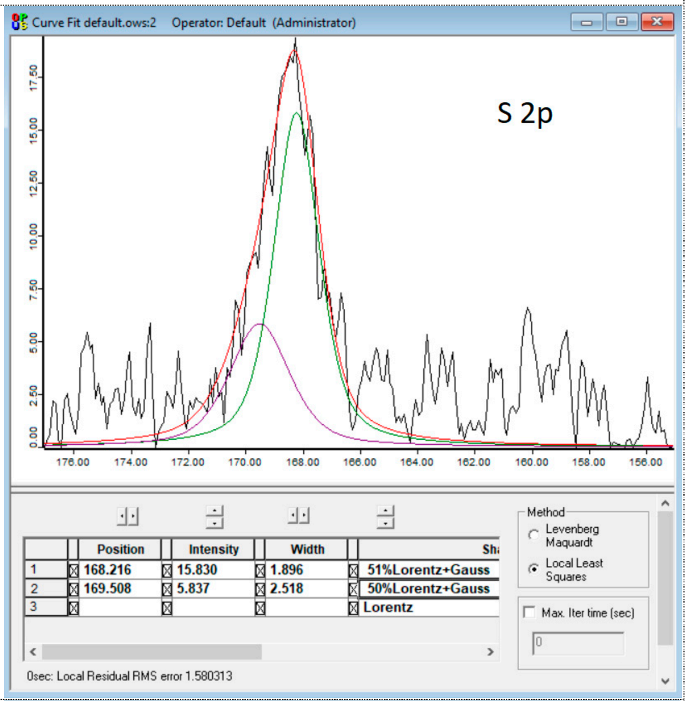	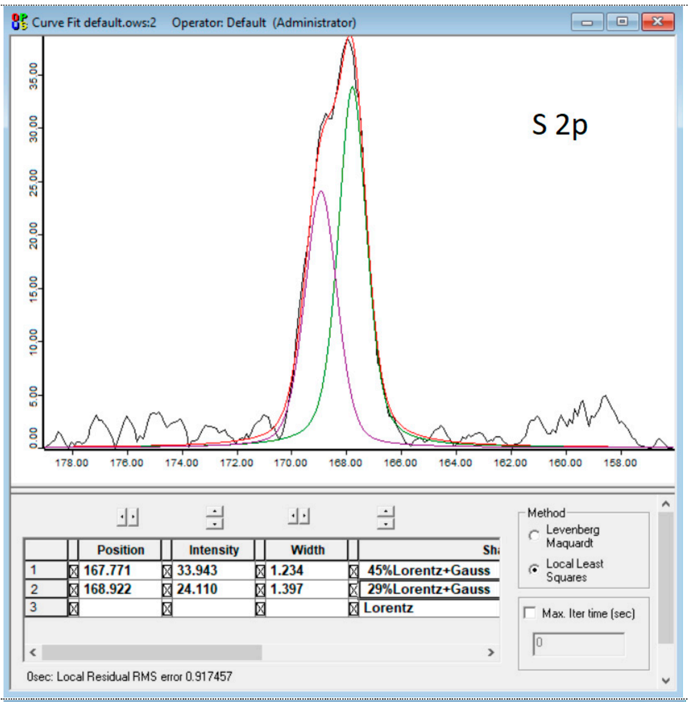
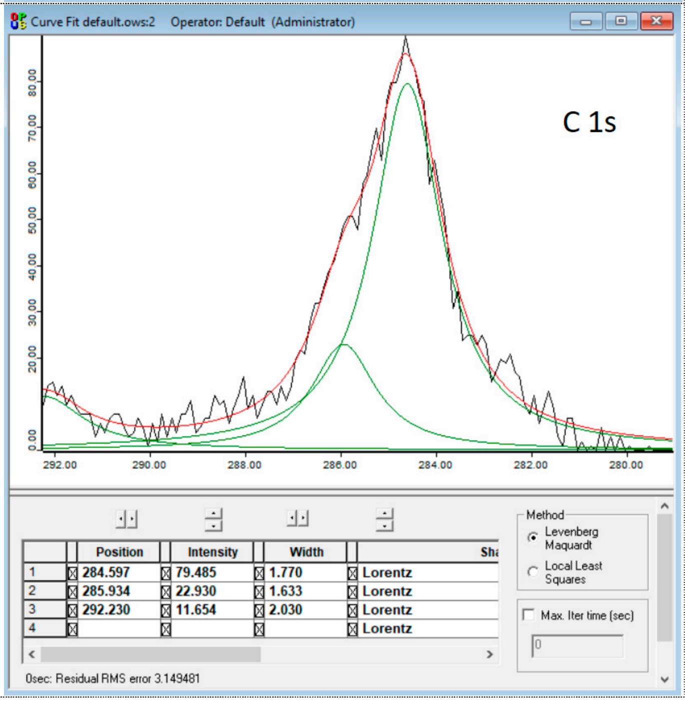	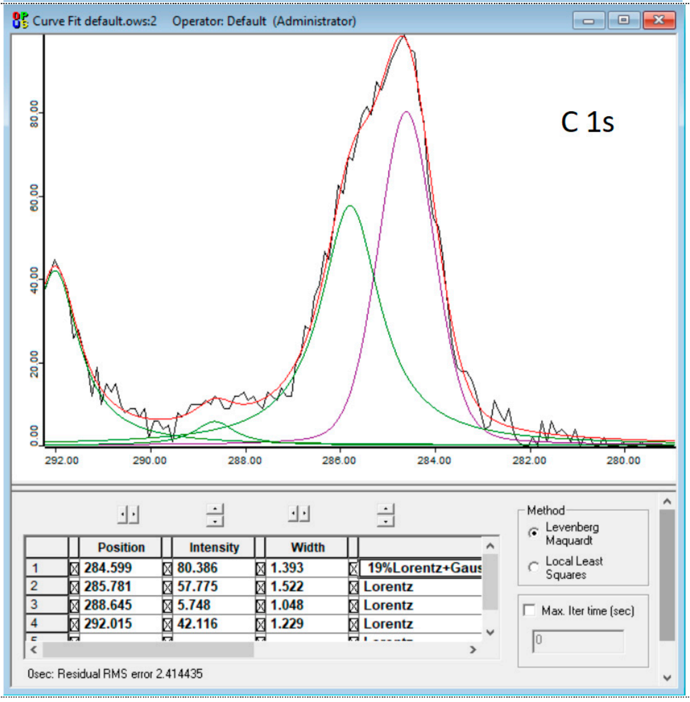

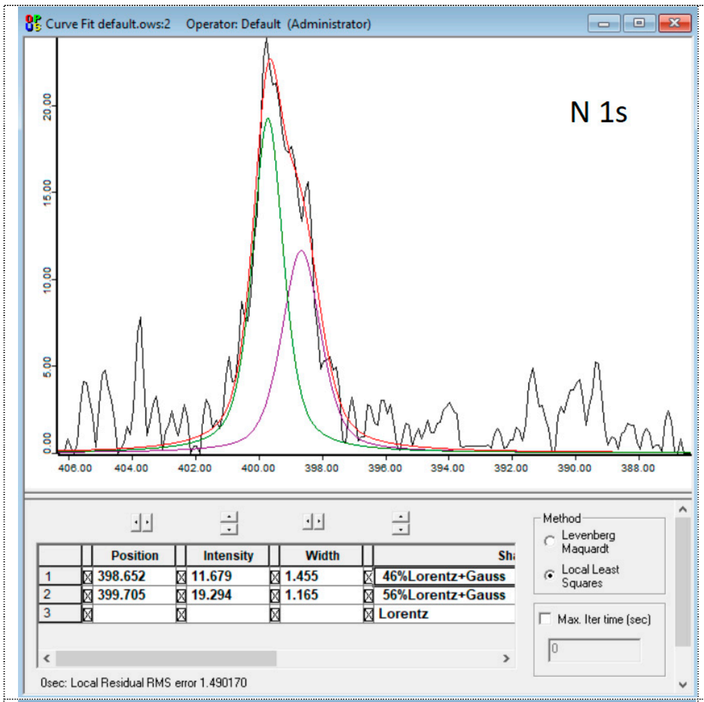
Curve Fit default.ows:2 Operator: Default (Administrator)
N 1s
20.00
15.00
10.00
5.00
0.00
406.00
404.00
402.00
400.00
398.00
396.00
394.00
392.00
390.00
388.00
Position
Intensity
Width
Sha
1 398.652 11.679 1.455 46%Lorentz+Gauss
2 399.705 19.294 1.165 56%Lorentz+Gauss
3 Lorentz
Method
Levenberg Maquardt
Local Least Squares
Max. Iter time (sec)
0sec: Local Residual RMS error 1.490170

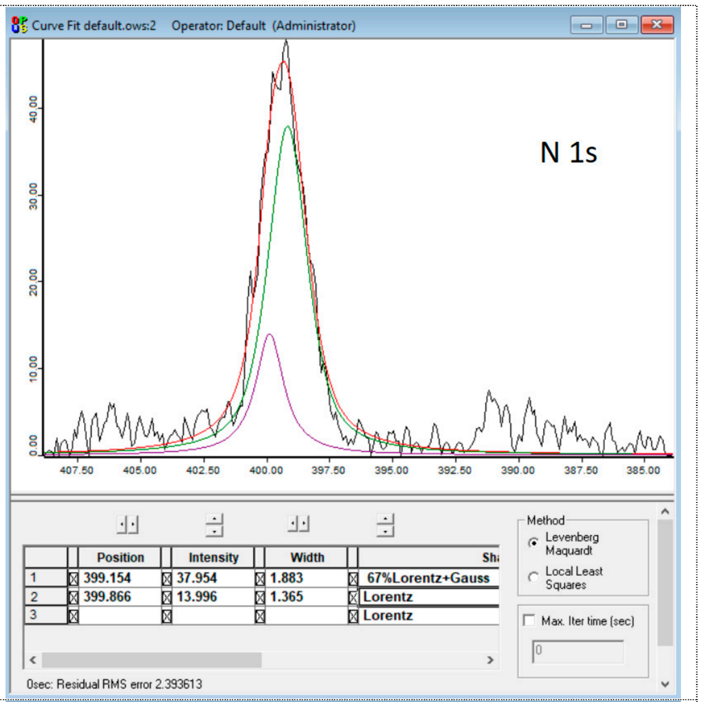
Curve Fit default.ows:2 Operator: Default (Administrator)
N 1s
40.00
30.00
20.00
10.00
0.00
407.50
405.00
402.50
400.00
397.50
395.00
392.50
390.00
387.50
385.00
Position
Intensity
Width
Sha
1 399.154 37.954 1.883 67%Lorentz+Gauss
2 399.866 13.996 1.365 Lorentz
3 Lorentz
Method
Levenberg Maquardt
Local Least Squares
Max. Iter time (sec)
0sec: Residual RMS error 2.393613

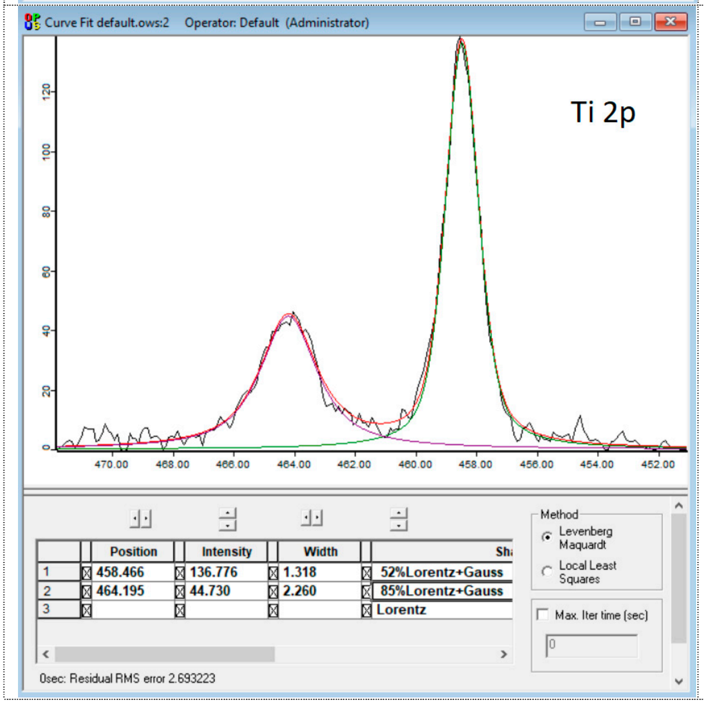
Curve Fit default.ows:2 Operator: Default (Administrator)
Ti 2p
120
100
80
60
40
20
0
470.00
468.00
466.00
464.00
462.00
460.00
458.00
456.00
454.00
452.00
Position
Intensity
Width
Sha
1 458.466 136.776 1.318 52%Lorentz+Gauss
2 464.195 44.730 2.260 85%Lorentz+Gauss
3 Lorentz
Method
Levenberg Maquardt
Local Least Squares
Max. Iter time (sec)
0sec: Residual RMS error 2.693223

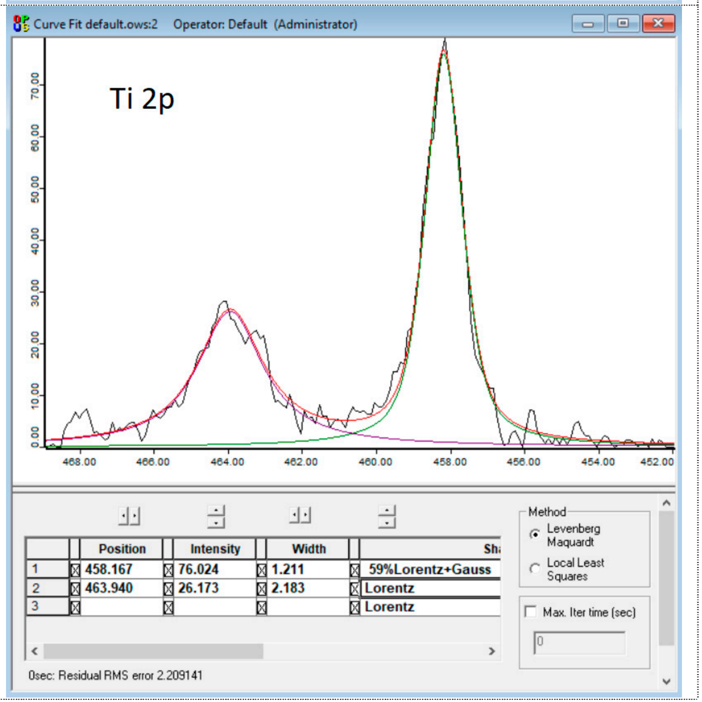
Curve Fit default.ows:2 Operator: Default (Administrator)
Ti 2p
70.00
60.00
50.00
40.00
30.00
20.00
10.00
0.00
468.00
466.00
464.00
462.00
460.00
458.00
456.00
454.00
452.00
Position
Intensity
Width
Sha
1 458.167 76.024 1.211 59%Lorentz+Gauss
2 463.940 26.173 2.183 Lorentz
3 Lorentz
Method
Levenberg Maquardt
Local Least Squares
Max. Iter time (sec)
0sec: Residual RMS error 2.209141

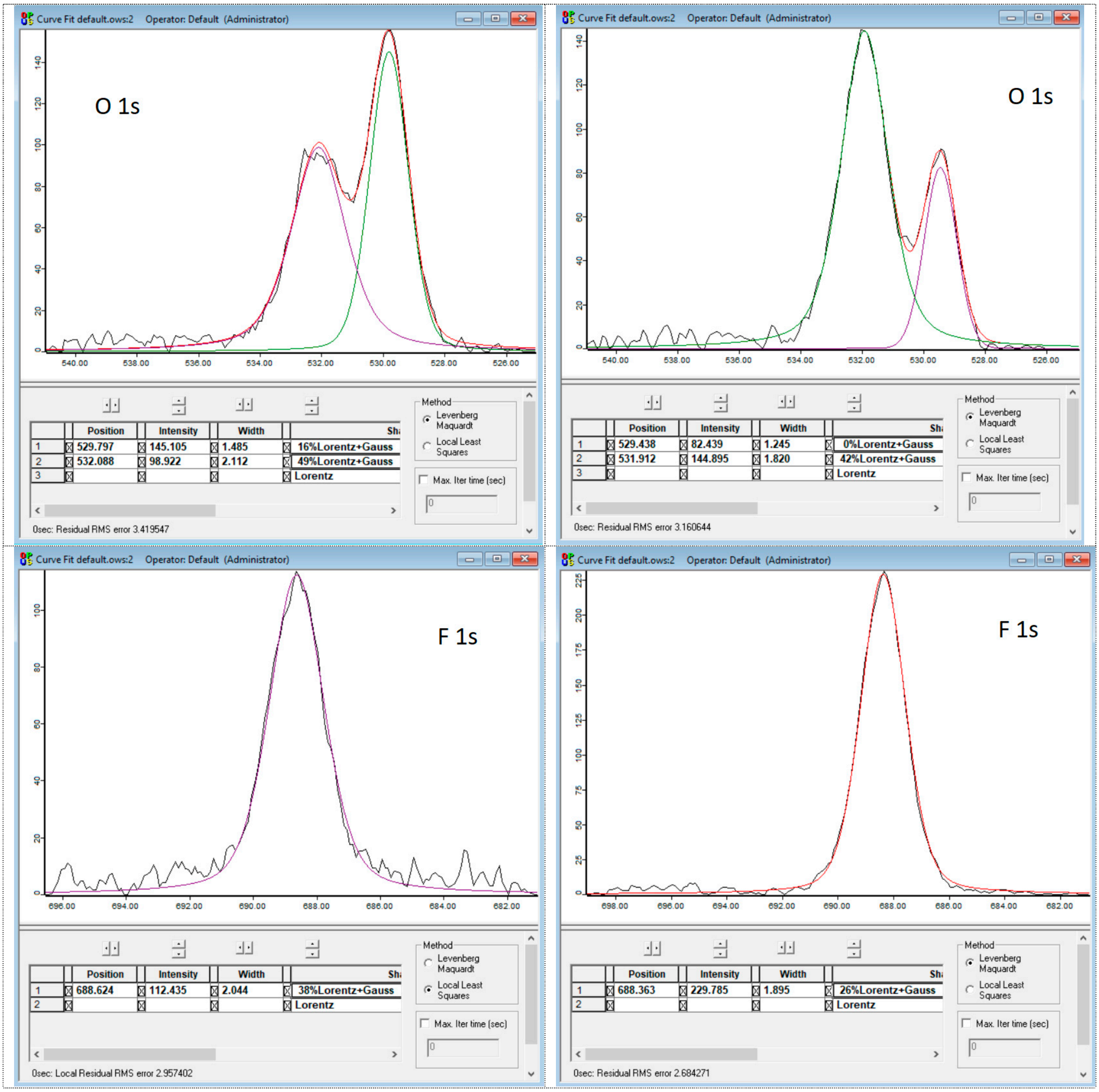

Figure S3. Best fits of XPS spectra of different elements of solid redox mediators (x = 0; X = TFSI$^-$ and Triflate$^-$). This figure also includes the best fits of XPS spectra of the Ti 2p element.

$x = 0.5$

X = $TFSI^{-}$ | X = $Triflate^{-}$

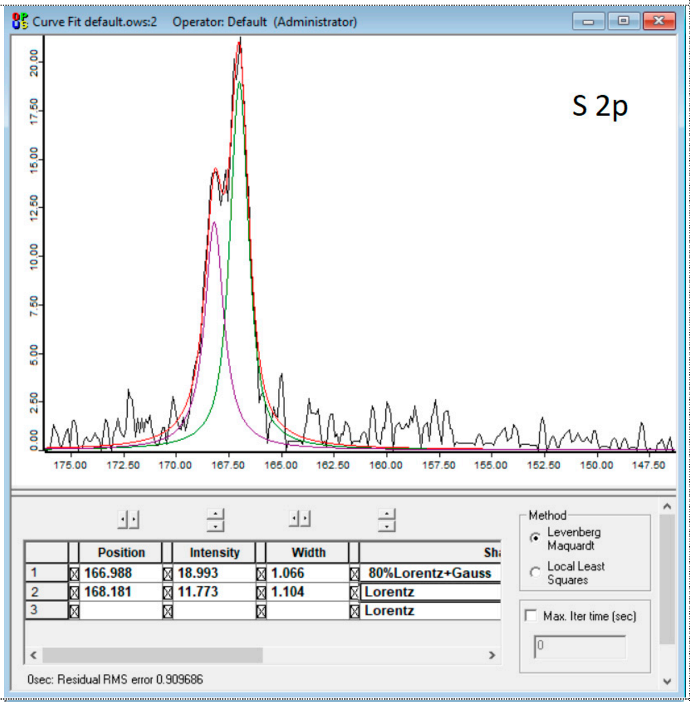

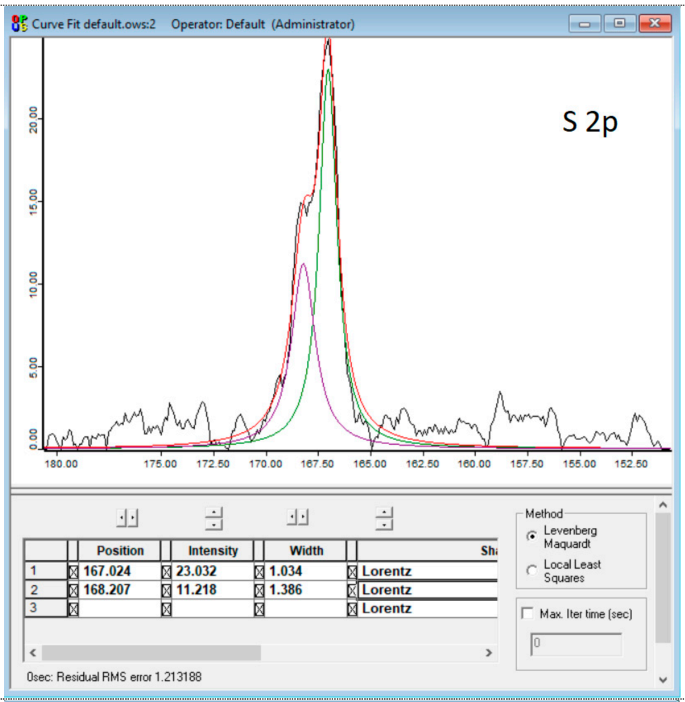

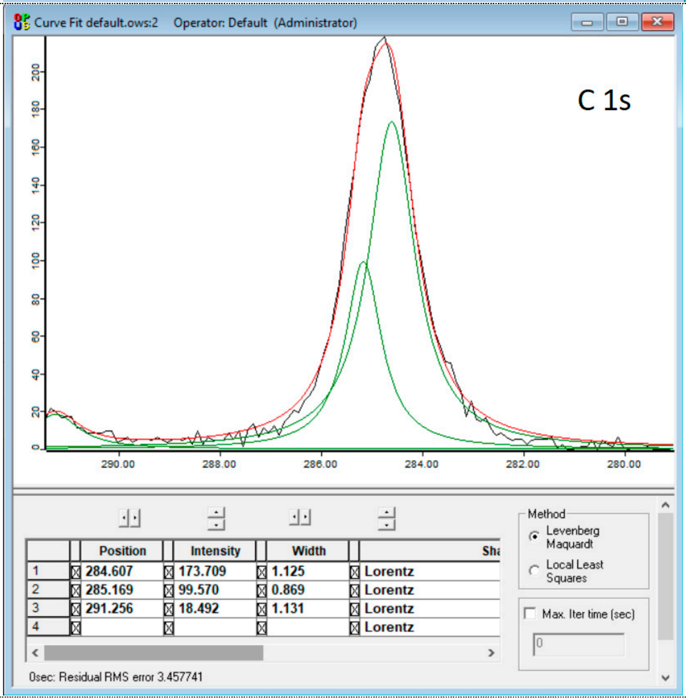

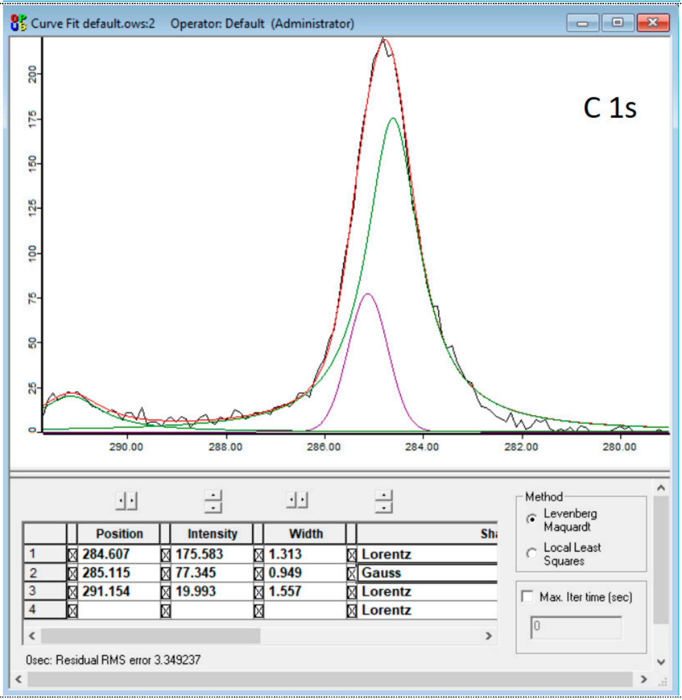

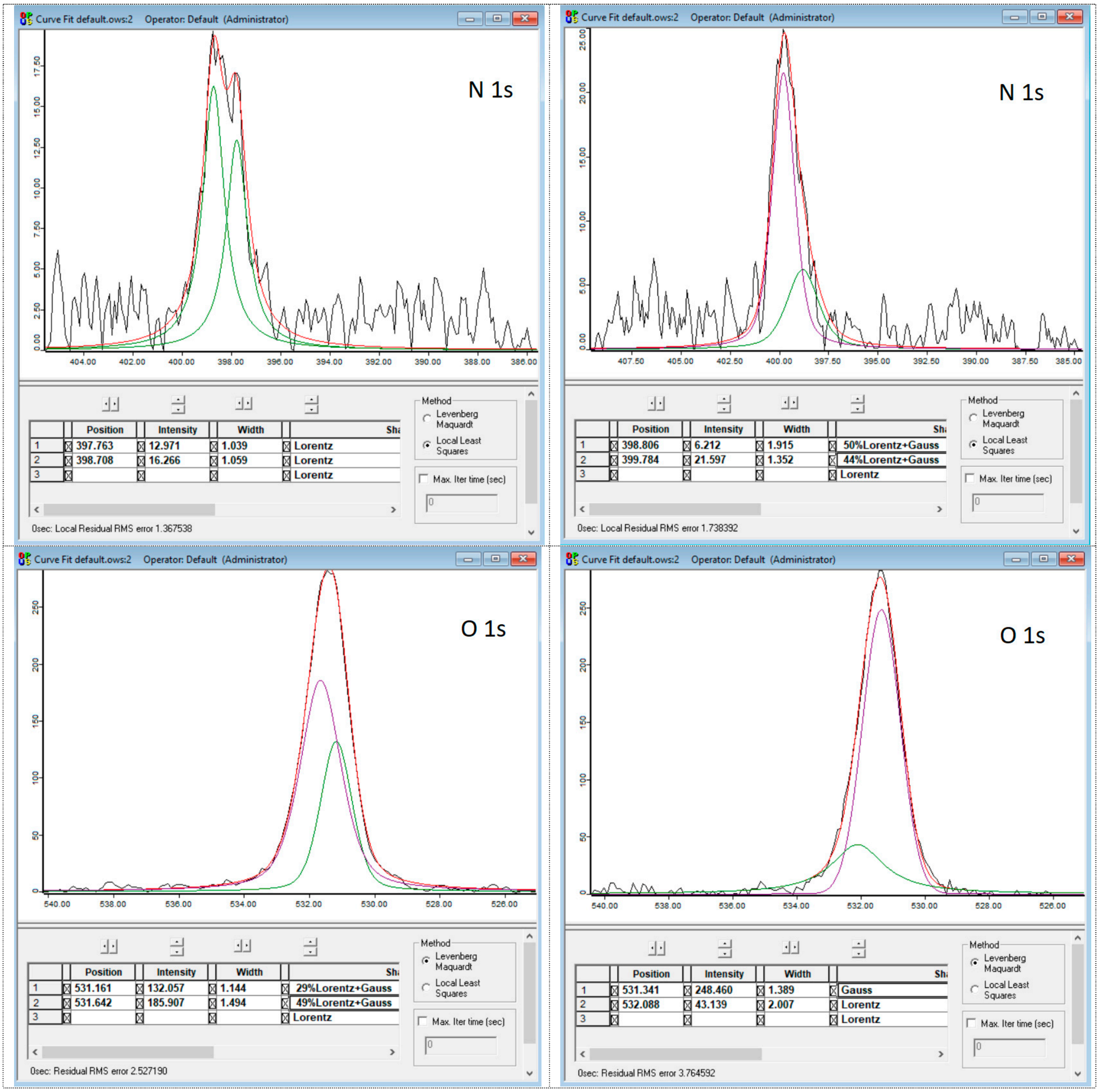
Curve Fit default.ows:2 Operator: Default (Administrator)
N 1s
Position | Intensity | Width | Sha
1 397.763 | 12.971 | 1.039 | Lorentz
2 398.708 | 16.266 | 1.059 | Lorentz
3 | | | Lorentz
Method
Levenberg Maquardt
Local Least Squares
Max. Iter time (sec)
0sec: Local Residual RMS error 1.367538
Curve Fit default.ows:2 Operator: Default (Administrator)
N 1s
Position | Intensity | Width | Sha
1 398.806 | 6.212 | 1.915 | 50%Lorentz+Gauss
2 399.784 | 21.597 | 1.352 | 44%Lorentz+Gauss
3 | | | Lorentz
0sec: Local Residual RMS error 1.738392
Curve Fit default.ows:2 Operator: Default (Administrator)
O 1s
Position | Intensity | Width | Sha
1 531.161 | 132.057 | 1.144 | 29%Lorentz+Gauss
2 531.642 | 185.907 | 1.494 | 49%Lorentz+Gauss
3 | | | Lorentz
0sec: Residual RMS error 2.527190
Curve Fit default.ows:2 Operator: Default (Administrator)
O 1s
Position | Intensity | Width | Sha
1 531.341 | 248.460 | 1.389 | Gauss
2 532.088 | 43.139 | 2.007 | Lorentz
3 | | | Lorentz
0sec: Residual RMS error 3.764592

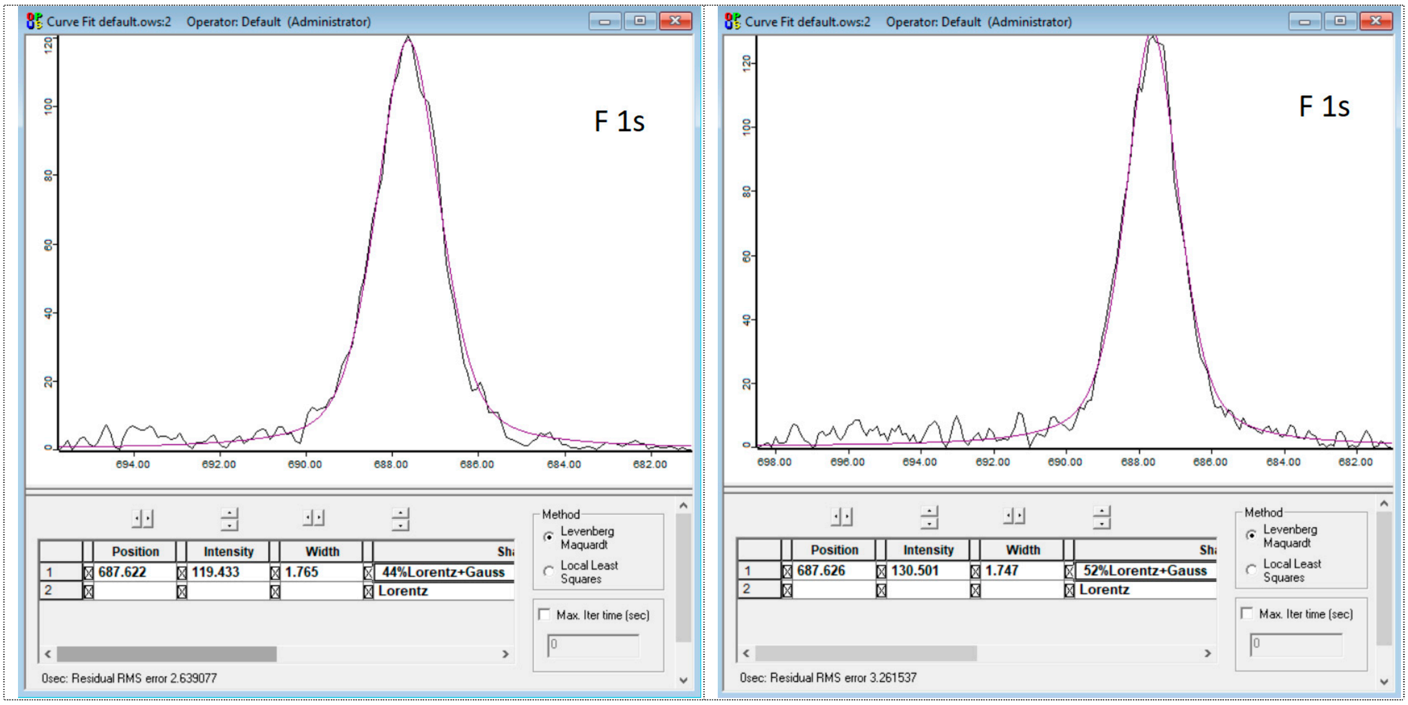

Figure S4. Best fits of XPS spectra of different elements of solid redox mediators (x = 0.5; X = TFSI$^-$ and Triflate$^-$).

$x = 1$

X = TFSI⁻	X = Triflate⁻
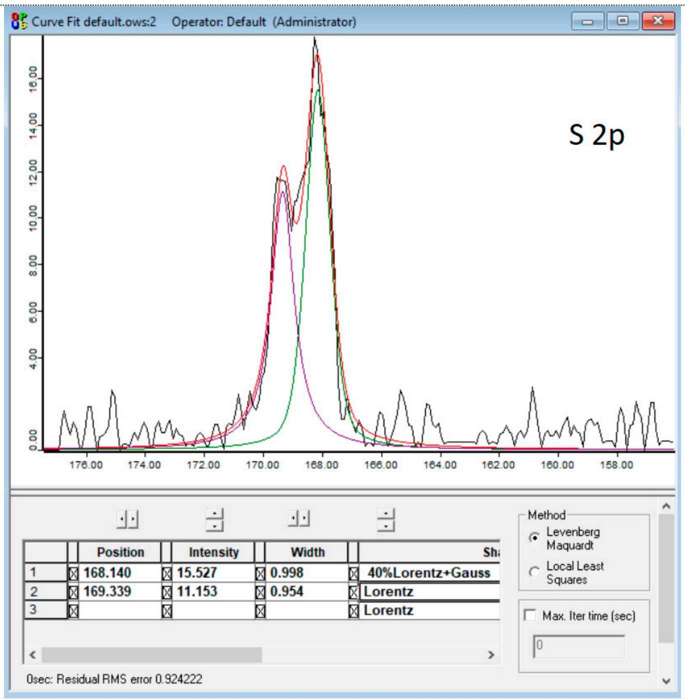	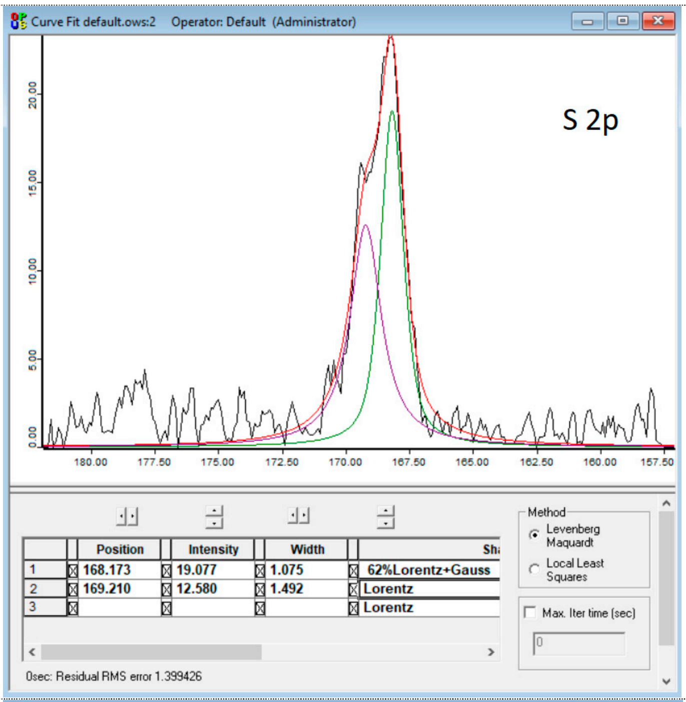
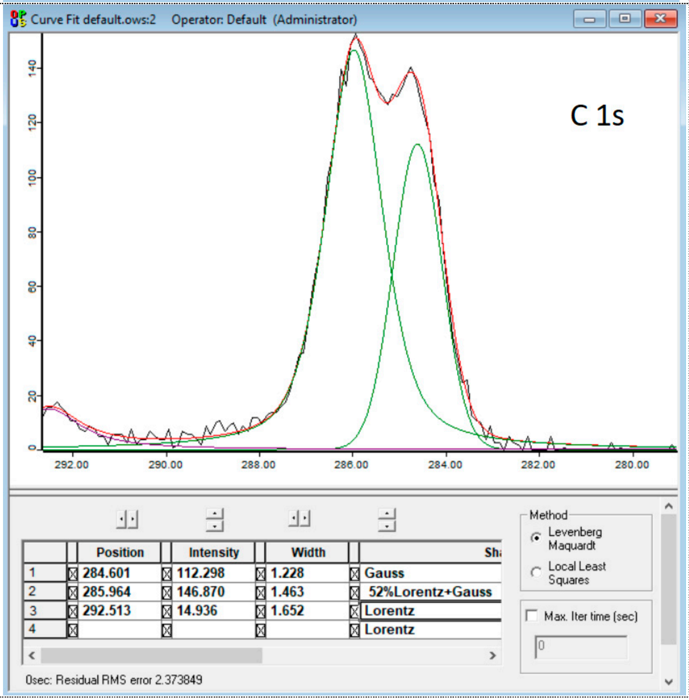	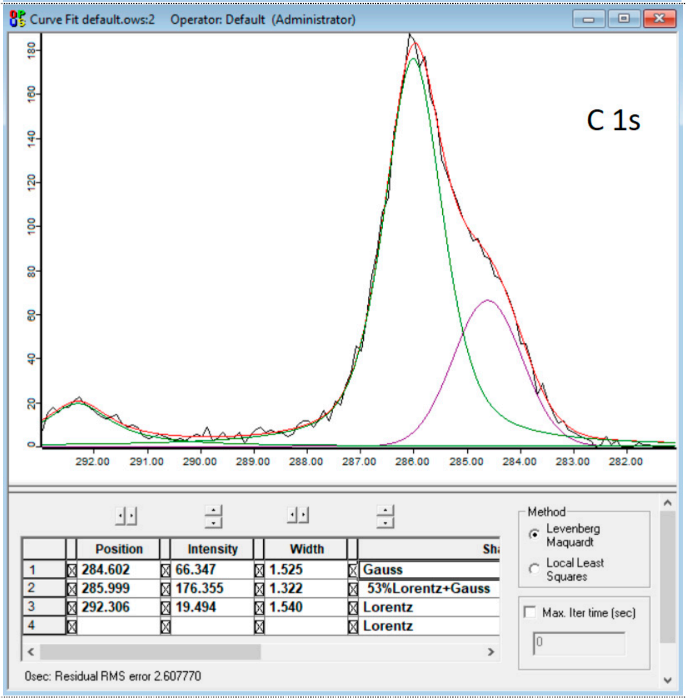

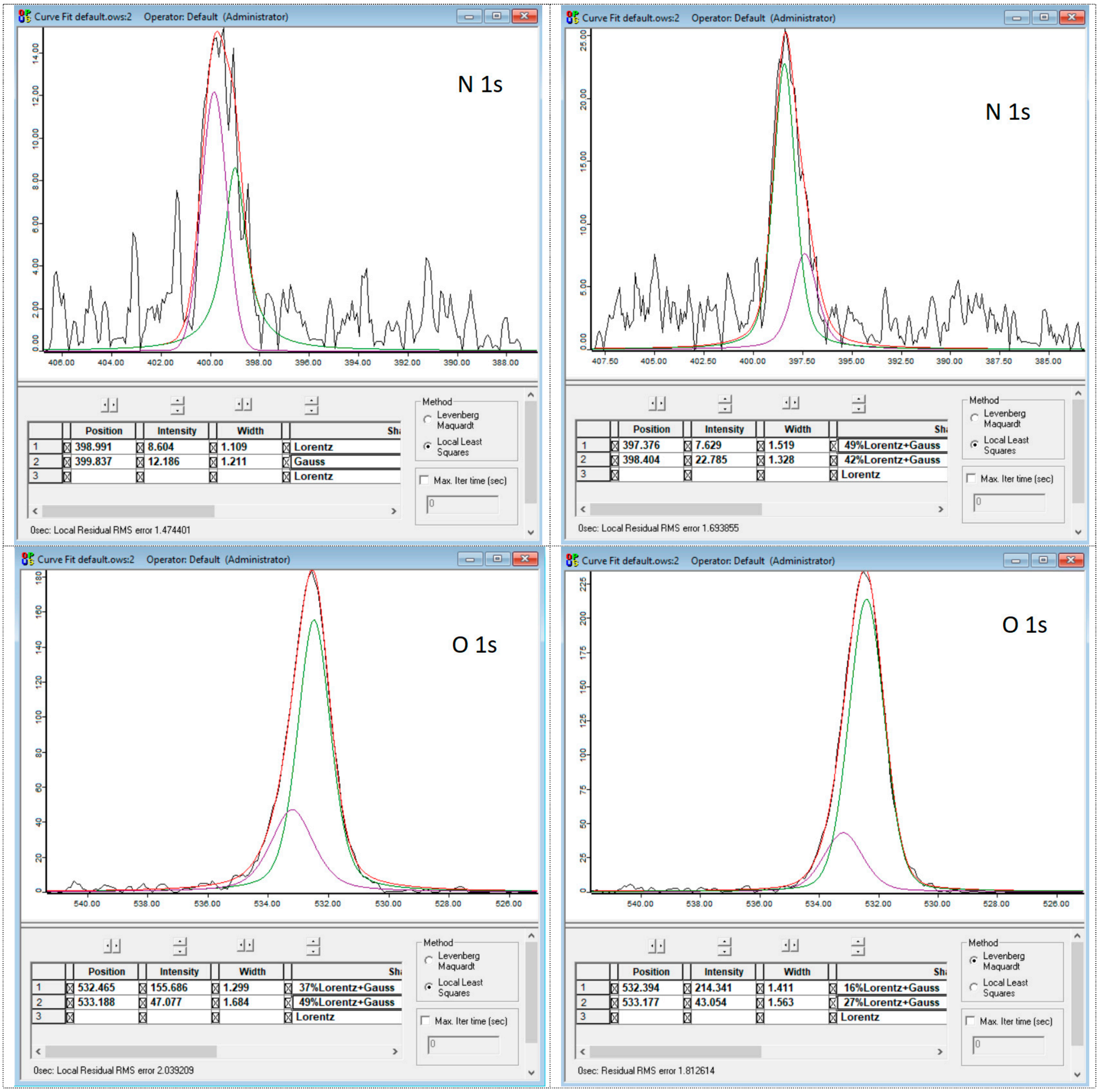
Curve Fit default.ows:2 Operator: Default (Administrator)
N 1s
Position Intensity Width Shape
1 398.991 8.604 1.109 Lorentz
2 399.837 12.186 1.211 Gauss
3 Lorentz
Method
Levenberg Maquardt
Local Least Squares
Max. Iter time (sec)
0sec: Local Residual RMS error 1.474401
Curve Fit default.ows:2 Operator: Default (Administrator)
N 1s
Position Intensity Width Shape
1 397.376 7.629 1.519 49%Lorentz+Gauss
2 398.404 22.785 1.328 42%Lorentz+Gauss
3 Lorentz
Method
Levenberg Maquardt
Local Least Squares
Max. Iter time (sec)
0sec: Local Residual RMS error 1.693855
Curve Fit default.ows:2 Operator: Default (Administrator)
O 1s
Position Intensity Width Shape
1 532.465 155.686 1.299 37%Lorentz+Gauss
2 533.188 47.077 1.684 49%Lorentz+Gauss
3 Lorentz
Method
Levenberg Maquardt
Local Least Squares
Max. Iter time (sec)
0sec: Local Residual RMS error 2.039209
Curve Fit default.ows:2 Operator: Default (Administrator)
O 1s
Position Intensity Width Shape
1 532.394 214.341 1.411 16%Lorentz+Gauss
2 533.177 43.054 1.563 27%Lorentz+Gauss
3 Lorentz
Method
Levenberg Maquardt
Local Least Squares
Max. Iter time (sec)
0sec: Residual RMS error 1.812614

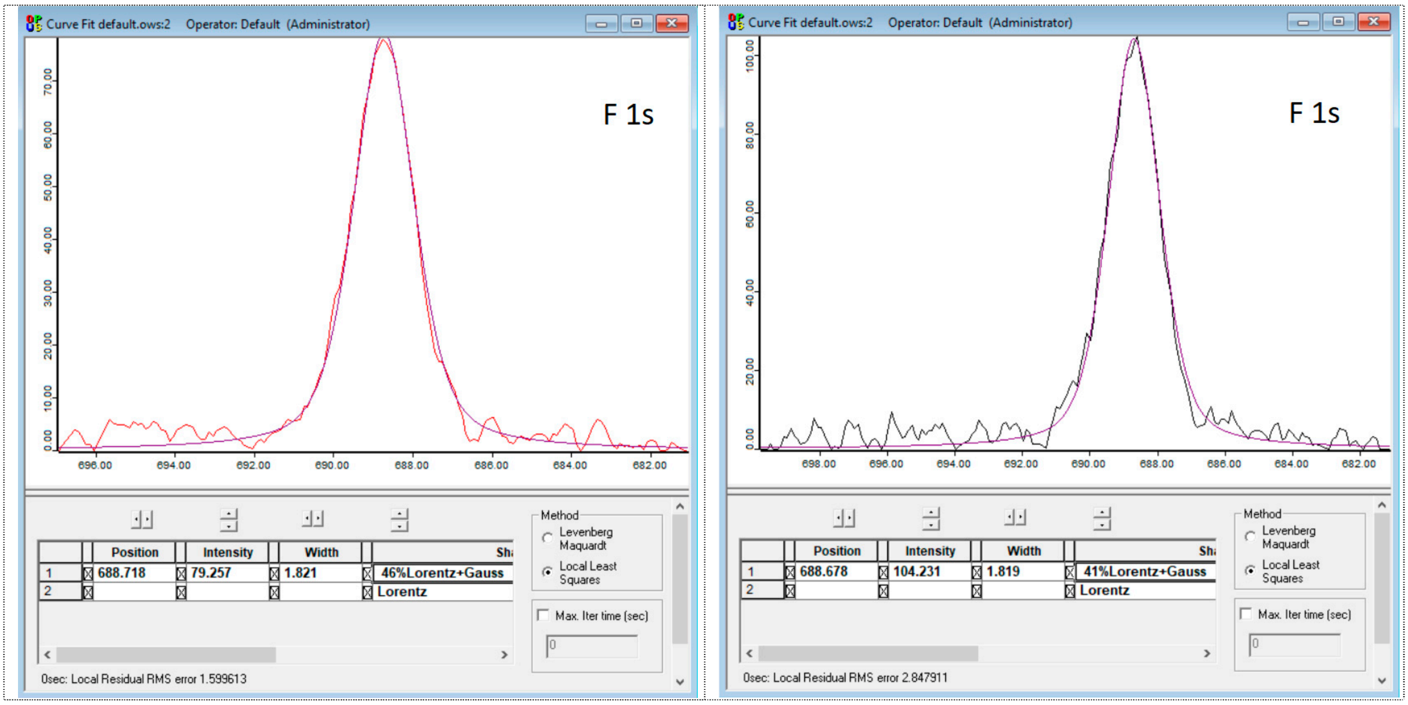

Figure S5. Best fits of XPS spectra of different elements of solid redox mediators (x = 1; X = TFSI$^-$ and Triflate$^-$).

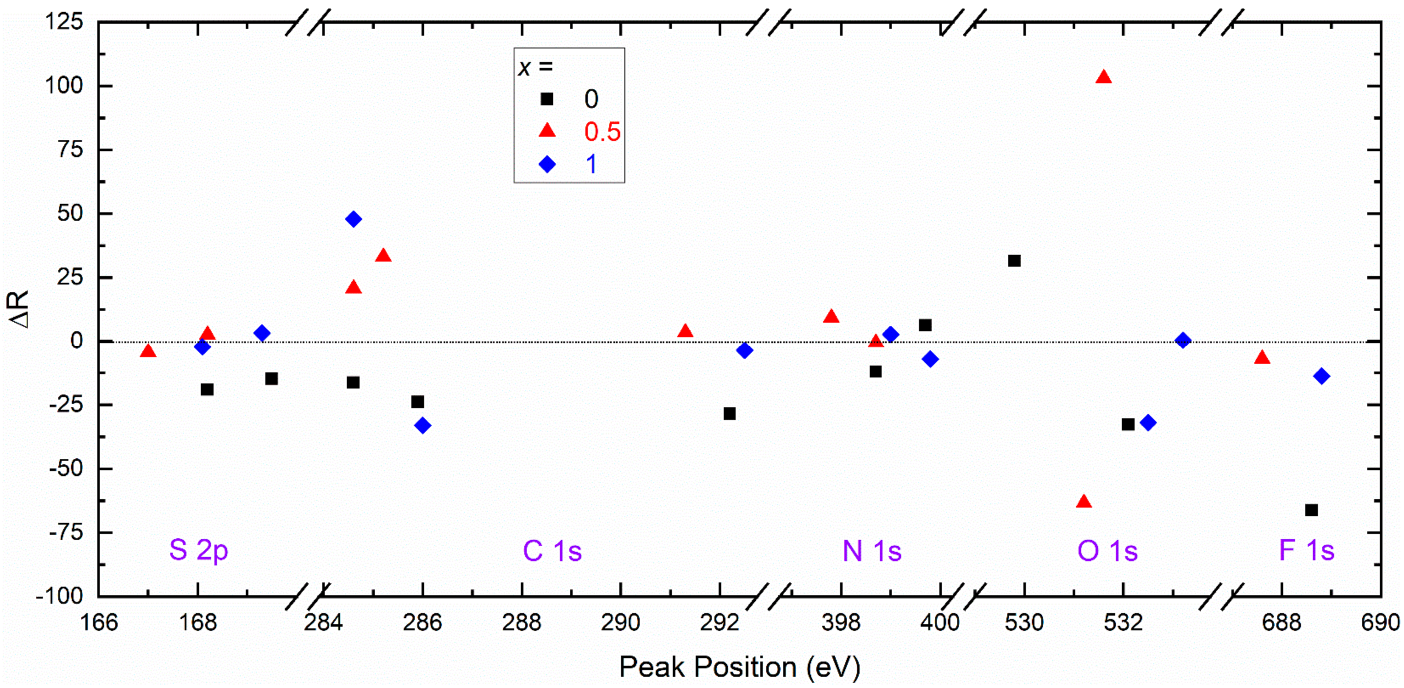

Figure S6. A plot of Δ*R* (= [intensity/width]TFSI – [intensity/width]Triflate) with peak position for different elements of solid redox mediators, [(1–*x*)SN: *x*PEO]-LiX-Co salts, where *x* = 0, 0.5, and 1. X = $TFSI^-$ and $Triflate^-$.

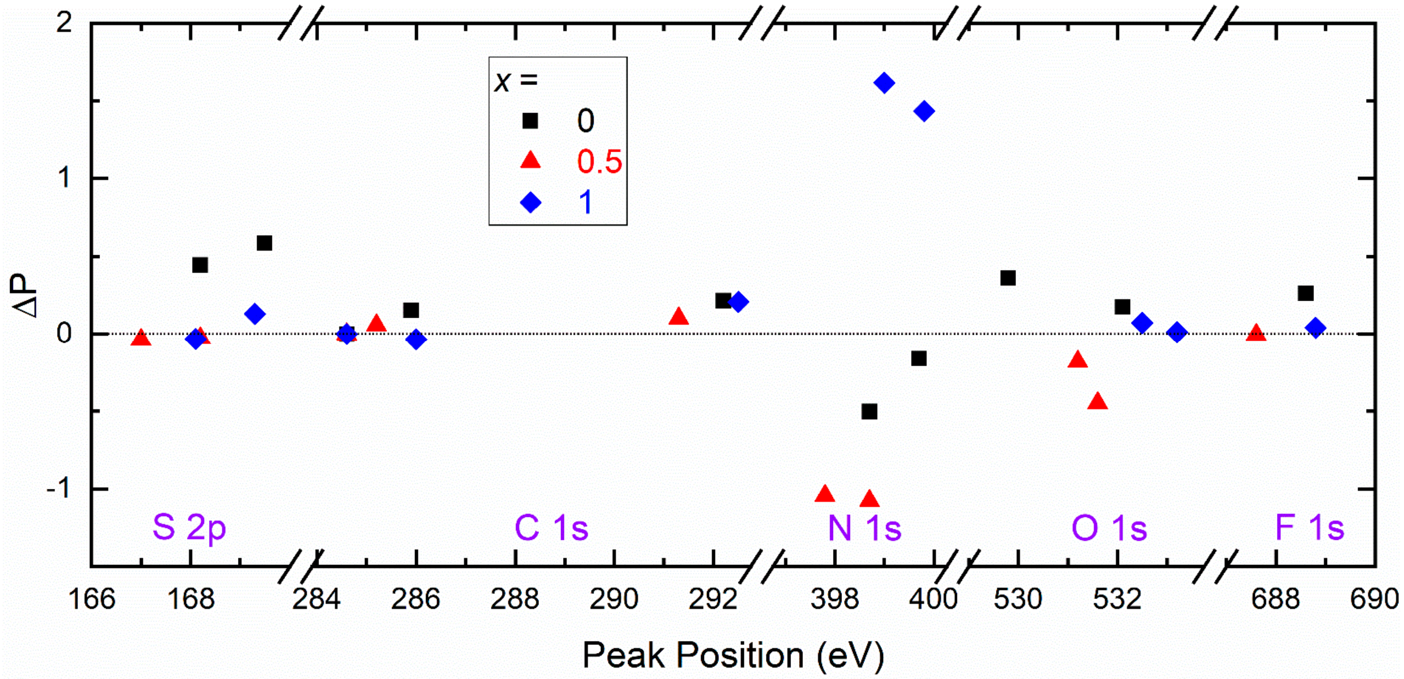

Figure S7. A plot of Δ*P* (= [peak position]TFSI – [peak position]Triflate) with peak position for different elements of solid redox mediators, [(1–*x*)SN: *x*PEO]-LiX-Co salts, where *x* = 0, 0.5, and 1. X = $TFSI^-$ and $Triflate^-$.
